# Systematic review and meta-analysis of prognostic models in Southeast Asian populations with acute myocardial infarction

**DOI:** 10.3389/fcvm.2022.921044

**Published:** 2022-07-26

**Authors:** Sophia Rasheeqa Ismail, Muhamad Khairul Nazrin Khalil, Mohd Shawal Faizal Mohamad, Shamsul Azhar Shah

**Affiliations:** ^1^Nutrition, Metabolic and Cardiovascular Research Centre, Institute for Medical Research, National Institutes of Health, Shah Alam, Malaysia; ^2^Department of Community Health, Faculty of Medicine, National University of Malaysia, Kuala Lumpur, Malaysia; ^3^Department of Cardiology, Hospital Canselor Tuanku Muhriz, Kuala Lumpur, Malaysia

**Keywords:** risk prediction model, prognostic model, acute myocardial infarction, validation, Southeast Asia

## Abstract

**Background:**

The cultural and genetic diversity of the Southeast Asian population has contributed to distinct cardiovascular disease risks, incidence, and prognosis compared to the Western population, thereby raising concerns about the accuracy of predicted risks of existing prognostic models.

**Objectives:**

We aimed to evaluate the predictive performances of validated, recalibrated, and developed prognostic risk prediction tools used in the Southeast Asian population with acute myocardial infarction (AMI) events for secondary events

**Methods:**

We searched MEDLINE and Cochrane Central databases until March 2022. We included prospective and retrospective cohort studies that exclusively evaluated populations in the Southeast Asian region with a confirmed diagnosis of an AMI event and evaluated for risk of secondary events such as mortality, recurrent AMI, and heart failure admission. The CHARMS and PRISMA checklists and PROBAST for risk of bias assessment were used in this review.

**Results:**

We included 7 studies with 11 external validations, 3 recalibrations, and 3 new models from 4 countries. Both short- and long-term outcomes were assessed. Overall, we observed that the external validation studies provided a good predictive accuracy of the models in the respective populations. The pooled estimate of the C-statistic in the Southeast Asian population for GRACE risk score is 0.83 (95%CI 0.72–0.90, *n* = 6 validations) and for the TIMI risk score is 0.80 (95%CI: 0.772–0.83, *n* = 5 validations). Recalibrated and new models demonstrated marginal improvements in discriminative values. However, the method of predictive accuracy measurement in most studies was insufficient thereby contributing to the mixed accuracy effect. The evidence synthesis was limited due to the relatively low quality and heterogeneity of the available studies.

**Conclusion:**

Both TIMI and GRACE risk scores demonstrated good predictive accuracies in the population. However, with the limited strength of evidence, these results should be interpreted with caution. Future higher-quality studies spanning various parts of the Asian region will help to understand the prognostic utility of these models better.

**Systematic review registration:**

https://www.crd.york.ac.uk/prospero/display_record.php?%20RecordID=228486.

## Introduction

Cardiovascular diseases (CVDs) are a major cause of disability and premature death globally—especially in low- and middle-income countries (1). In Southeast Asia, the burden of CVD has been reported to be increasing throughout the region even in rural and urban settings of low-income countries such as Cambodia and Myanmar (2–5). Patients having a recent acute myocardial infarction (AMI) event, a common presentation of coronary artery disease, are at a higher risk of secondary events such as recurrent AMI, heart failure, and even mortality (6). Despite the global improvements in prevention and healthcare, the prevalence of major adverse cardiovascular events (MACE) has been increasing with several trends in Southeast Asia being worse than in other regions (7–9). Amongst the determinants of MACE, ethnicity is an important contributor to the relationship between risk factors and coronary artery disease severity (10). Pre-eminently, evidence on risk factors of MACE in Asian populations has been conflicting (10–13). This highlights the importance of using locally adapted strategies and locally validated tools for better prevention strategies to improve survival and quality of life.

Usage of CVD risk prediction models in clinical medicine is important for stratifying risks in individuals to allow for a more personalized, and eventually cost-effective treatment. Thus far, the vast majority of the CVD prediction models were derived and validated in Western populations- with only a small number validated in the Southeast Asian population (14). The cultural and genetic diversity of the Southeast Asian population has contributed to distinct CVD risks, incidence, and prognosis compared to the Western population (15–17), thereby raising concerns about under-or overestimation of the predicted outcomes (18–20). Therefore, it is pivotal to evaluate the accuracy of prognostic models for CVD for appropriate secondary events prevention and control strategies. In this study, we aimed to systematically evaluate the predictive performances of prognostic risk prediction tools used in the Southeast Asian population with an AMI event for MACE and to explore the predictive performances of recalibrated and newly developed prognostic risk models for the Southeast Asian population with an AMI event.

## Methods

### Study design

This systematic review was conducted in accordance with the Critical Appraisal and Data Extraction for Systematic Reviews of Prediction Modeling Studies (CHARMS) Checklist (21) and reported in accordance with the Preferred Reporting Items for Systematic Reviews and Meta-Analyses (PRISMA) checklist (22) (Supplementary File 1). The protocol of this systematic review was registered with PROSPERO (Registration number: CRD42021228486). The registered protocol is available at https://www.crd.york.ac.uk/prospero/display_record.php?RecordID=228486.

### Eligibility criteria

We included prospective and retrospective cohort studies that exclusively evaluated populations from any of the following countries in the Southeast Asia regions, such as Malaysia, Singapore, Brunei, Indonesia, Myanmar, Vietnam, Thailand, Indonesia, Cambodia, Laos, and Timor-Leste. Participants with a confirmed diagnosis of an AMI event such as ST-segment myocardial infarction (STEMI), non-ST segment myocardial infarction, or unspecified type of AMI were included. We included both single endpoints and composite endpoints that reported any MACE. This included all-cause mortality, cardiac-related mortality, any type of recurrent AMI, admission for heart failure, and stroke. Outcomes developed both in-hospital and following discharge were included with no restrictions in timelines. Prognostic risk models included were either externally validated models (defined as the assessment of previously developed models in a new setting, new timeline, or study population than that of the derivation cohort), recalibrated models (defined as adjustments to the model equation of a previously developed model to a new setting or study population), or a newly developed model (defined as the development of a new prognostic model from a new setting or study population). We included any type of multivariable prognostic models (e.g., Cox proportional hazards models and logistic regression models). We excluded studies that did not evaluate predictive measures such as calibration and discrimination in their validation study.

### Search databases

We searched for potential articles in MEDLINE and Cochrane Central (which included PubMed, EMBASE, CINAHL, Clinicaltrials.gov and ICTRP registry) until 21 March 2022. A manual search for additional relevant studies and review articles using references from retrieved articles was also performed. A detailed description of our search strategy is available in Supplementary File 2. No restriction in language was applied.

### Selection of studies, data extraction, and management

Two authors (SRI and MKNK) independently screened the titles and/or abstracts for potentially eligible studies, and then independently evaluated the full text of the shortlisted articles to determine eligibility. We outlined the study selection process in a PRISMA diagram. Two authors (SRI and MKNK) independently extracted all data from each included study using a standardized data collection form. We developed the data collection form for this review by incorporating the items in the CHARMS Checklist (21) and other items from a similar review (23). This included the source of data, participants (eligibility criteria, recruitment method, and description), outcomes (type and definition of outcomes), candidate predictors (number, type, definition, and handling of predictors), sample size (number of outcomes, events per variable), missing data (number and handling of missing data), model development (if applicable), model performance (calibration, discrimination, and classification measures), model evaluation, and results. All data recorded were checked for accuracy by another review author. Disagreements along any of these steps were resolved by discussion (e.g., inclusion and exclusion of unsure articles), with the input of a third author (MSFM or SAS) when necessary.

### Assessment of the risk of bias

We used the Prediction model study Risk of Bias Assessment Tool (PROBAST) to assess the risk of bias (ROB) of the included studies (24). The PROBAST includes the following four steps: (1) Specification of the systematic review question(s); (2) Classification of the type of prediction model evaluation; (3) Assessment of risk of bias and applicability; (4) Overall judgment. In the ROB assessment, four domains were evaluated as follows: (1) Participants; (2) Predictors; (3) Outcome; (4) Analysis. Each domain was judged either low, high, or unclear ROB. We used the published guidelines by the PROBAST authors as a guiding tool for our ROB assessment (25). Signaling questions were rated as yes (Y), probably yes (PY), probably no (PN), no (N), or no information (NI). Two review authors (SRI and MKNK) assessed each of the included studies for risk of bias independently (Supplementary File 3). Disagreements along these steps were resolved *via* discussion (e.g., differences in judgments of a particular domain between SRI and MKNK), with the input of a third author (SAS) when necessary.

### Predictive performance of models

We compared each prognostic model using the following three different methods: (1) Discrimination; (2) Calibration, and (3) Reclassification, if applicable. Discrimination is the ability of a prediction model to differentiate between two outcome classes. In this review, we evaluated the discriminative ability of the model between those with and without the MACE. For binary outcomes, discrimination is generally presented as the area under the receiver operating characteristic curve (AUC), or the concordance statistics (C-statistic) (26). C-statistic ranges between 0 and 1 with 0.5 being defined as random concordance and 1 as a perfect concordance (26). Calibration reflects the goodness-of-fit of the model between the observed outcomes and predictors. For binary outcomes, the most used calibration measure is the Hosmer-Lemeshow goodness-of-fit test (26). The Hosmer-Lemeshow test is reported with a *p*-value, in which a *p*-value < 0.05 is considered a poor calibration of the model (26). Reclassification assesses the improvement in prediction from using a new predictor in addition to existing predictors. Reclassification measures are generally presented as the Net Reclassification Index (NRI). The NRI is reported as the percentage of increment of each of the defined event categories. We had no restriction on the type of statistical tests for the measurement of the predictive performance of each included model.

### Evidence synthesis

We initially performed a descriptive analysis approach of all the included studies and categorized by the type of model assessment as follows: externally validated models, recalibrated models, and newly developed models. Subsequently, descriptive and quantitative assessments of the studies were performed using the model. When the same prognostic model was evaluated in multiple validation studies, we calculated the pooled estimate of the predictive performance of these studies through a random-effects meta-analysis that considered any between-study heterogeneity. We evaluated the pooled predictive performance through the discrimination measure of C-statistic. The sample size for each population was deemed to be adequate if there were at least 10 events per candidate predictor (27, 28). All analyses, including the meta-analysis, were performed using R version 4.0.1 (R Foundation for Statistical Computing, Vienna, Austria) (Supplementary File 4).

## Results

**Figure 1:**
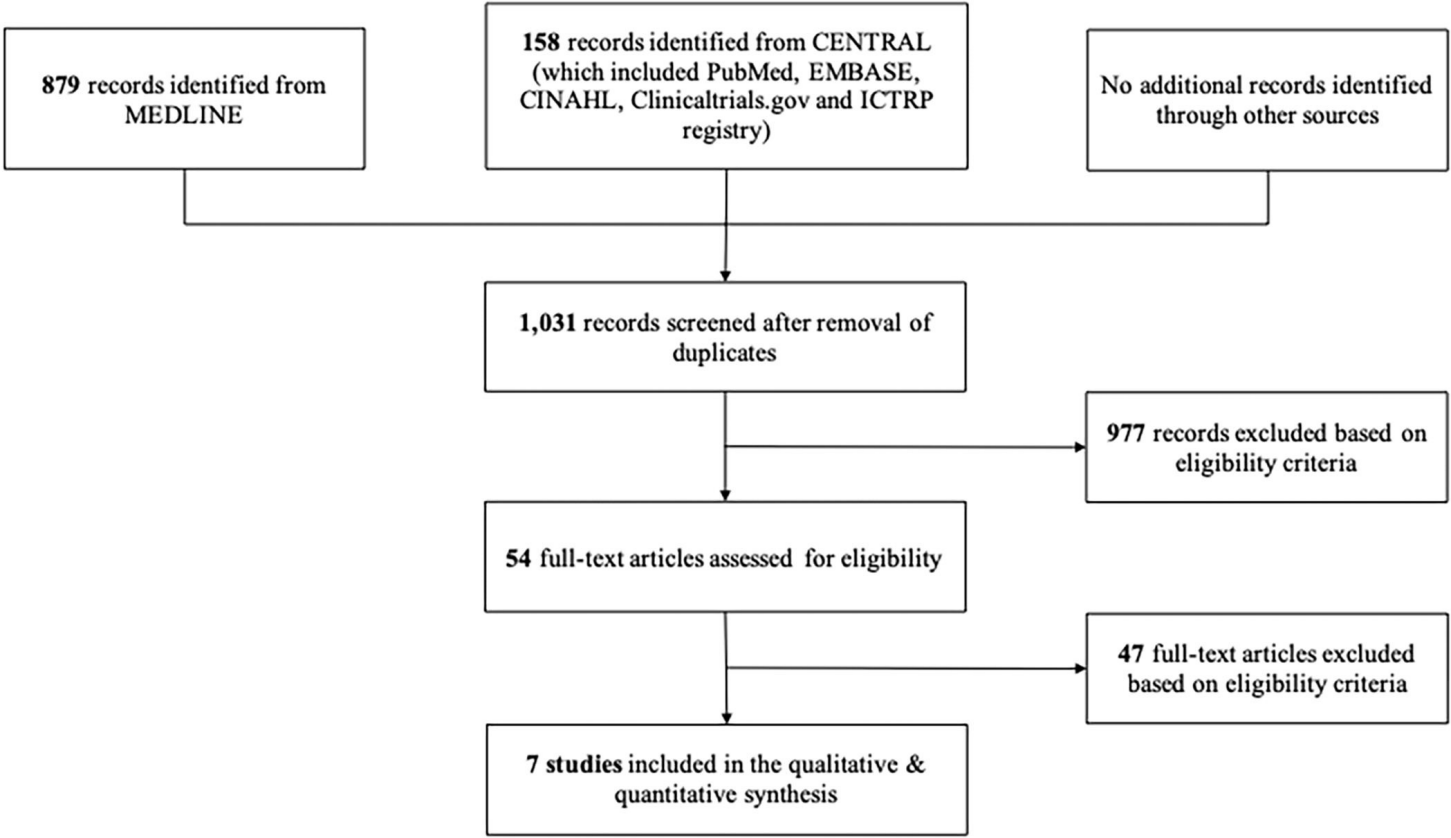
PRISMA flow diagram.

Our search yielded 1,037 records and 977 records were excluded after titles and abstracts screening. A total of 54 full-text articles were further excluded based on eligibility criteria. Notably, seven studies were eligible for assessment in this review. The PRISMA flow diagram of the study identification processes is shown in Figure 1.

The included studies assessed prognostic models in populations from Indonesia (*n* = 1) (29), Malaysia (*n* = 2) (30, 31), Singapore (*n* = 2) (32, 33), and Thailand (*n* = 2) (34, 35). The total number of participants in the included studies ranged from 152 to 15,151 participants with a median of 4,701 participants. Notably, five out of seven studies included participants from multicenter registries and ethnicity subgroup group analysis was performed in one study (33). All studies included only local participants and were retrospective in nature. Index disease was STEMI in six studies while the remaining study included the unspecified type of AMI. Outcomes assessed were mortality (*n* = 7), composite cardiovascular outcomes (*n* = 2), and hospitalization for heart failure (*n* = 1). Duration of follow-up included the duration of index hospitalization (*n* = 4), 30-day of index event (*n* = 5), 6-month of index event (*n* = 2), and 1-year of the index event (*n* = 1).

Validated risk scores were the GRACE score (29, 33–35) and TIMI score (29–31) only. The GRACE score had six external validations from four studies and three recalibrations from one study. The TIMI risk score was validated for in-hospital mortality in two studies, for 30-day mortality in two studies, and for 1-year mortality in one study. All existing models that were developed outside of the Southeast Asia region were evaluated by different authors than the original model's authors except for one study (33). Recalibration of an existing prognostic model was performed only for the GRACE score in one study (33). Prognostic models developed with the local population were available in three studies (30, 32, 33). Out of the three newly developed models, one utilized a machine-learning approach for model development. The overall characteristics of the included studies are summarized in Table 1.

**Table 1:** Overall characteristics of the included studies.

**Study name, country**	**Model name**	**Population cohort**	**Timeline**	**Sample size**	**Eligibility criteria**	**Index disease**	**Endpoint(s)**
External validation							
Aziz et al. (30), Malaysia	TIMI score	Malaysia National Cardiovascular Disease Database	2006 to 2016	12,368	All patients from the ACS registry without exclusion were used including patients who received reperfusion (fibrinolysis, PPCI), angiography demonstrating spontaneous reperfusion, or CABG) for STEMI	STEMI	In-hospital mortality, 30-day mortality, and 1-year mortality
Chan et al. (33), Singapore	GRACE score	Singapore Myocardial Infarction Registry	2000 to 2005	15,151	Patients with AMI were identified for inclusion. Patients younger than 21 years, who were non-residents, and who died within 24 h of admission were excluded.	AMI	In-hospital mortality
Chotechuang et al. (34), Thailand	GRACE score	Maharaj Nakorn Chiang Mai Hospital STEMI Registry	2007 to 2012	152	The post-fibrinolytic therapy STEMI patients who underwent a delayed coronary intervention (24 h to 2 weeks) were included. Patients were excluded if they failed fibrinolytic therapy, performed an early coronary intervention (<24 h), underwent very long delayed coronary intervention, refused further interventions after fibrinolytic therapy, underwent PPCI or rescue PCI, or had previous history of CABG.	STEMI	Composite CV outcomes at 1-month and 6-month. Composite outcomes included all-cause mortality, re-hospitalization with ACS, re-hospitalization with heart failure, and stroke
Chotechuang et al. (35), Thailand	GRACE score	Maharaj Nakorn Chiang Mai Hospital STEMI Registry	2007 to 2012	341	The post-fibrinolytic therapy STEMI patients who underwent a delayed coronary intervention (24 h to 2 weeks) were included. Patients were excluded if they failed fibrinolytic therapy, performed an early coronary intervention (<24 h), underwent very long delayed coronary intervention, refused further interventions after fibrinolytic therapy, underwent PPCI or rescue PCI, or had previous history of CABG.	STEMI	Composite CV outcomes at 30-day and 6-month. Composite outcomes included death, re-hospitalization with ACS, re-hospitalization with heart failure, and stroke
Martha et al. (29), Indonesia	GRACE Score	Dr. Hasan Sadikin General Hospital Bandung, Indonesia	July 2018 to June 2019	255	Patients diagnosed with STEMI or with the ICD code of I21.0-I21.3. Patients with I21.0-I21.3 code but with a diagnosis other than STEMI, such as NSTEACS and occlusion myocardial infarction, and those with incomplete or absent medical records, were excluded.	STEMI	In-hospital mortality
Martha et al. (29), Indonesia	TIMI Score	Dr. Hasan Sadikin General Hospital Bandung, Indonesia	July 2018 to June 2019	255	Patients diagnosed with STEMI or with the ICD code of I21.0-I21.3. Patients with I21.0-I21.3 code but with a diagnosis other than STEMI, such as NSTEACS and occlusion myocardial infarction, and those with incomplete or absent medical re- cords, were excluded.	STEMI	In-hospital mortality
Selvarajah et al. (31), Malaysia	TIMI score	Malaysia National Cardiovascular Disease Database	2006 to 2009	4,701	Registered patients who presented with STEMI	STEMI	30 days mortality
Newly developed models							
Aziz et al. (30), Malaysia	SVMvarImp-SBE-SVM	Malaysia National Cardiovascular Disease Database	2006 to 2016	12,368	All patients from the ACS registry without exclusion were used including patients who received reperfusion (fibrinolysis, PPCI), angiography demonstrating spontaneous reperfusion, or urgent CABG) for STEMI	STEMI	In-hospital mortality, 30-day mortality, and 1-year mortality
Bulluck et al. (32), Singapore	SMIR	Singapore Myocardial Infarction Registry	2008 to 2015	8,082	Patients presenting to the hospital with a STEMI within 12 h of symptoms onset were reperfused by PPCI. Patients with a STEMI but not reperfused by PPCI or those with an LBBB were excluded	STEMI	In-hospital cardiac mortality, 30-day cardiac mortality, 1-year cardiac mortality, 1-year hospitalization for heart failure
Chan et al. (33), Singapore	Singapore score	Singapore Myocardial Infarction Registry	2000 to 2005	15,151	Patients with AMI were identified for inclusion. Patients younger than 21 years, who were non-residents, and who died within 24 h of admission were excluded.	AMI	In-hospital mortality
Recalibrated models							
Chan et al. (33), Singapore	Recalibrated GRACE score	Singapore Myocardial Infarction Registry	2000 to 2005	15,151	Patients with AMI were identified for inclusion. Patients younger than 21 years, who were non-residents, and who died within 24 h of admission were excluded.	AMI	In-hospital mortality

*ACS, acute coronary syndrome; AMI, Acute myocardial infarction; CABG, coronary artery bypass graft; CV, cardiovascular; LBBB, left bundle branch block; NSTEACS, non-ST elevation acute coronary syndrome; PCI, percutaneous coronary intervention; PPCI, primary percutaneous coronary intervention; STEMI, ST segment elevation myocardial infarction*.

### GRACE score

**Figure 2:**
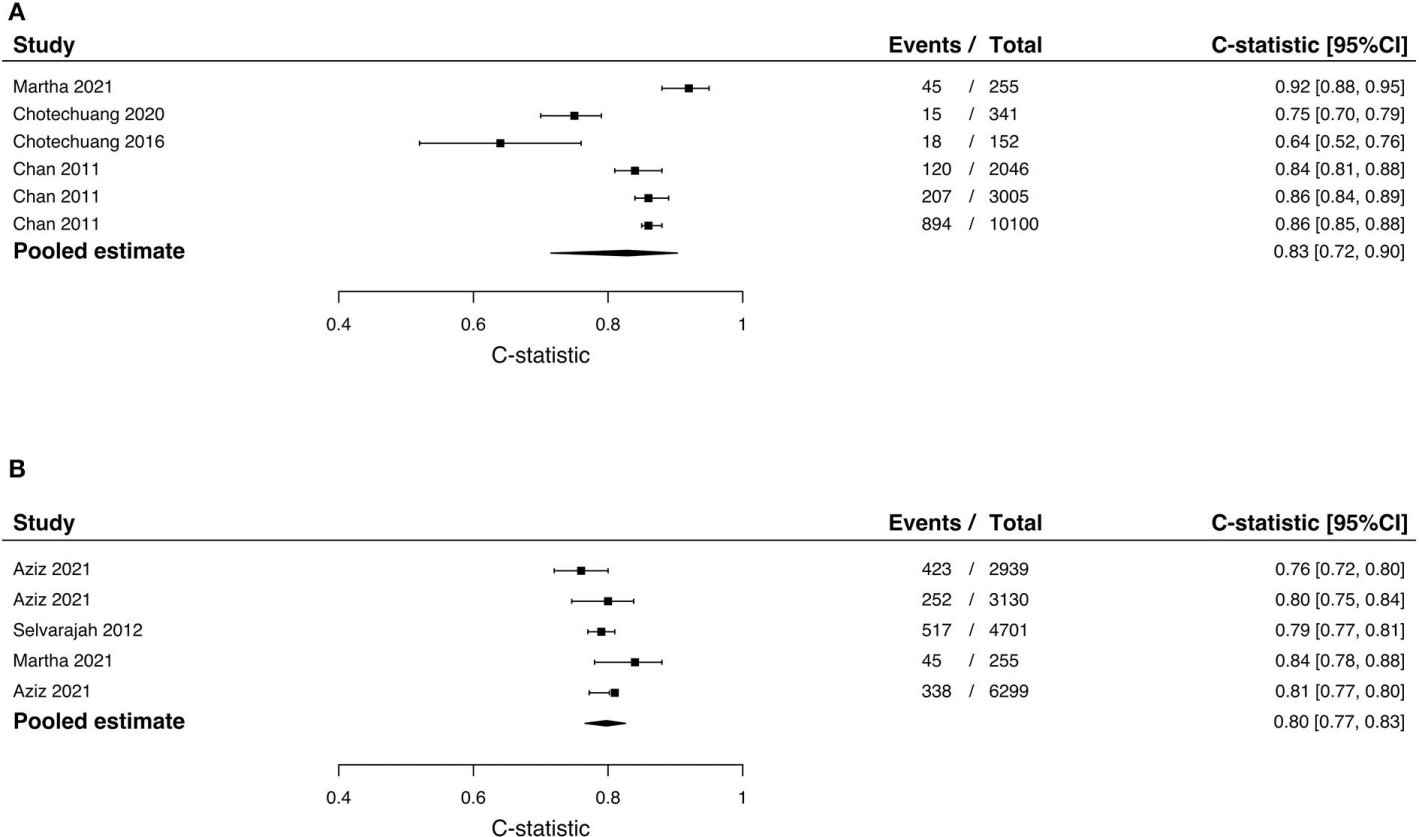
Meta-analysis of C-statistic for the **(A)** GRACE score and for the **(B)** TIMI risk score.

The GRACE score was validated in three populations as follows: Indonesia (29), Thailand (34, 35), and Singapore (33). Only one study had multicenter recruitment of participants (33). Notably, the two studies from Thailand included participants from the same center and had similar outcomes; however, the number of events and number of participants were different. In-hospital mortality was evaluated in the Malay Singapore, Chinese Singapore, Indian Singapore, and Indonesian populations while 6-month composite cardiovascular outcomes were evaluated in the Thai population in two studies. Adequate events-to-predictor was only achieved in one study (three validations) (33). Discrimination was measured in all validation studies while calibration was only reported in three validation studies. Overall, the GRACE score performed fairly well in all populations with C-statistic ranging between 0.64 and 0.92. The score performed best in the Indonesian population, however, this study was underpowered. The pooled estimate of the C-statistic in the Southeast Asian population is 0.83 (95%CI 0.72–0.90) (Figure 2A). Calibration was only performed in one study (three validations) and was not significant (33). A summary of predictive performance for the GRACE score external validation studies is reported in Table 2.

**Table 2:** Summary of predictive performances of included prognostic models.

**Model**	**Outcome**	**Study name**	**Population**	**Events**	**Calibration measure** ^*^	Discrimination measure
External validation
GRACE score	In-hospital mortality	Chan et al. (33)	Singapore (Chinese)	894/ 10100	NS	0.86 (0.85–0.88)
			Singapore (Malay)	207/ 3005	NS	0.86 (0.84–0.89)
			Singapore (Indian)	120/ 2046	NS	0.84 (0.81–0.88)
		Martha et al. (29)	Indonesia	45/255	Not reported	0.92 (0.88–0.95)
	6-month composite CV	Chotechuang et al. (34)	Thailand	18/ 152	Not reported	0.64 (0.52–0.76)
		Chotechuang et al. (35)	Thailand	15/341	Not reported	0.75 (0.70–0.79)
TIMI score	In-hospital mortality	Aziz et al. (30)	Malaysia	252/ 3130	Not reported	0.81 (0.77–0.80)
		Martha et al. (29)	Indonesia	45/ 255	Not reported	0.84 (0.78–0.88)
	30-day mortality	Aziz et al. (30)	Malaysia	252/ 3130	Not reported	0.80 (0.75–0.84)
		Selvarajah et al. (31)	Malaysia	517/ 4701	NS	0.79 (0.77–0.81)
	1-year mortality	Aziz et al. (30)	Malaysia	423/ 2939	Not reported	0.76 (0.72–0.80)
Recalibration
Recalibrated GRACE score	In-hospital mortality	Chan et al. (33)	Singapore (Chinese)	894/ 10100	NS	0.86 (0.85–0.88)
			Singapore (Malay)	207/ 3005	NS	0.86 (0.84–0.89)
			Singapore (Indian)	120/ 2046	NS	0.84 (0.81–0.88)
New model
SVMvarImp-SBE-SVM	In-hospital mortality	Aziz et al. (30)	Malaysia	252/ 3130	Not reported	0.88 (0.85–0.91)
	30-day mortality		Malaysia	252/ 3130	Not reported	0.90 (0.87–0.94)
	1-year mortality		Malaysia	423/ 2939	Not reported	0.84 (0.80–0.87)
SMIR	In-hospital cardiac mortality	Bulluck et al. (32)	Singapore	741/ 11546	Not reported	0.92 (0.91–0.93)
	30-day cardiac mortality		Singapore	780/ 11546	Not reported	0.90 (0.89–0.92)
	1-year cardiac mortality		Singapore	956/ 11546	Not reported	0.88 (0.87–0.90)
	1-year hospitalization for heart failure		Singapore	399/ 11546	Not reported	0.87 (0.85–0.89)
Singapore score	In-hospital mortality	Chan et al. (33)	Singapore (Chinese)	894/ 10100	Significant	0.88 (0.87–0.90)
			Singapore (Malay)	207/ 3005	NS	0.89 (0.87–0.91)
			Singapore (Indian)	120/ 2046	NS	0.88 (0.84–0.91)

*CV, cardiovascular; NS, not significant. The number of events is presented as the total number of events/ total number of participants. Discrimination measures are all reported in C-statistic value (95%CI)*.

* *Calibration measures are reported as not significant (NS) if Hosmer Lemeshow is >0.05 or significant if Hosmer Lemeshow is < 0.05*.

Recalibration of the GRACE score to the Singapore population was performed by retaining the regression coefficients from the original GRACE score and by substituting the original GRACE score constants with the constants unique to their population cohort (33). Ethnic-specific models were reported for the Chinese, Malay, and Indian populations. The predictive performance of the recalibrated models was assessed for the risk of in-hospital mortality. Adequate events-to-predictor was achieved in all three recalibrated models. Calibration assessment was not significant in all three models. Discrimination assessed was the lowest for the Indian subgroup (C-statistic 0.84; 95%CI 0.81–0.88) while C-statistic was approximate in the Malay and Chinese models. Overall, there was no difference in predictive measures between the original GRACE score and the recalibrated GRACE score for the Singaporean population. A summary of predictive performance for recalibrated GRACE scores is reported in Table 2.

### TIMI score

The TIMI score for STEMI was validated in three studies and two populations, i.e., in Malaysia and Indonesia. Outcomes assessed were in-hospital mortality (*n* = 2), 30-day mortality (*n* = 2), and 1-year mortality (*n* = 1). For the risk of in-hospital mortality, discrimination of the score was better in the Indonesian population (C-statistic 0.84; 95%CI 0.78–0.88) but adequate events-to-predictor was not achieved in this validation. For the risk of 30-day mortality, the discriminative measures were approximate in the two validation studies and adequate events-to-predictor was achieved in both validation studies. For the risk of 1-year mortality, the TIMI risk score performed well in the validated population. The pooled estimate of the C-statistic in the Southeast Asian population for the TIMI score is 0.80 (95%CI: 0.77–0.83) (Figure 2B). Calibration was only performed in one validation and was insignificant (31). A summary of predictive performance for recalibrated GRACE scores is reported in Table 2.

### Newly developed models

In this review, we evaluated the following five developed models: the SVMvarImp-SBE-SVM model had three different models for different outcomes, SMIR, and Singapore Score. Although Aziz and colleagues included several other model options, the SVMvarImp-SBE-SVM model was the best performing model (30). The derivative cohort populations were the STEMI population from the Malaysia National Cardiovascular Disease Database for the SVMvarImp-SBE-SVM model and the STEMI population from Singapore Myocardial Infarction Registry for the SMIR and Singapore score. The number of predictors included varied between 8 and 15 predictors. Only the following two predictors were constant across the five newly developed models: age and Killip class. Other predictors could be grouped into either prescribed medications, blood investigations, cardiac investigations/interventions, or past medical history. All new models achieved adequate events-to-predictors' sample size.

The SVMvarImp-SBE-SVM model was the best performing model under the machine-learning approach for the evaluation of the risk of mortality during hospitalization, at 30-day and 1-year mortality (30). Supervised classification machine-learning algorithms used were Logistic Regression, Support Vector Machine, and Random Forest. The number and list of predictors for the SVMvarImp-SBE-SVM model varied according to the outcome. The model for in-hospital mortality included 15 predictors. The model for 30-day mortality included 13 predictors. The model for 1-year mortality included 12 predictors. The list of predictors included is summarized in Supplementary File 5. Of the three models, the discriminative measure was the highest for 30-day mortality (C-statistic 0.90; 95%CI 0.87–0.94) and the lowest for 1-year mortality (C-statistic 0.84; 95%CI 0.80–0.87). Calibration was not reported in all three models. The NRI was the highest for the in-hospital model (20%) followed by 30-day (19%) and 1-year mortality (14%). A summary of predictive performance for SVMvarImp-SBE-SVM models is reported in Table 2.

The SMIR evaluated cardiac mortality during hospitalization, at 30-day and 1-year as well as 1-year hospitalization for heart failure. The model included 9 predictors and was both internally and externally validated. Internal validation was performed through bootstrapping techniques on the derivation cohort. Although calibration was not performed for SMIR, discrimination of the model performed better in the validation cohort (C-statistic 0.903; 95%CI 0.882–0.923) than in the derivation cohort (C-statistic 0.881; 95%CI 0.867–0.896). Reclassification was not reported for this model. The misclassification rate was however reported to assess the discrimination power of the final model: 14.0% for in-hospital cardiac mortality, 14.7% for 30-day cardiac mortality, 16.2% for 1-year cardiac mortality, and 24.0% for 1-year hospitalization for heart failure. A summary of predictive performance for SMIR is reported in Table 2.

The Singapore Score was ethnicity-specific and had different regression coefficients and weightage for Chinese, Malay, and Indian groups. The model included 8 predictors. No external validation was performed for this new model. The model had good and similar discrimination for all three ethnicities. However, there was good calibration for the Malay and Indian groups (*p* = 0.514 and *p* = 0.586, respectively) and poor calibration for the Chinese group (*p* < 0.002). A summary of predictive performance for the Singapore score is reported in Table 2.

### Risk of bias of the included studies

**Figure 3:**
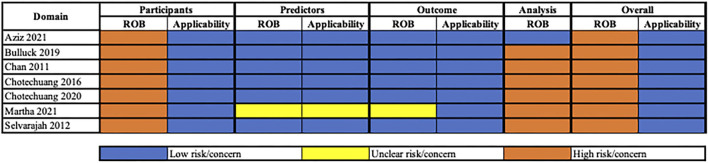
Summary of the risk of bias assessment.

We evaluated the risk of bias in each of the included studies with the PROBAST tool. The overall risk of bias and applicability assessment is presented in Figure 3. Briefly, all seven studies were assessed as relatively higher risk of bias but with low concerns of applicability. The studies were evaluated as high risk in the participant's domain due to the usage of registry data and retrospective in nature. Registry data are often not collected for the sole purpose of development, validation, and updating of prediction models thereby limiting prespecified and consistent methods for valid data recording (25). Sensitivity analysis for the type of source data could not be performed as all the included studies were classified as relatively higher risk of bias in the participants' domain. Notably, six out of seven studies were evaluated as having a relatively higher risk of bias in the analysis domain due to improper handling of missing data and/or an insufficient number of events per predictor. One study had insufficient details for the ROB assessment of predictors and outcomes. An insufficient number of events per predictor were judged as a relatively higher risk of bias due to its resulting large standard error and confidence interval leading to higher inaccuracies in the measurement (25). In contrast, improper handling of missing data (e.g., simply excluding enrolled participants with any missing data from the analysis) may lead to biased predictor–outcome associations and biased model performance. As only one study was judged as low risk of bias in the analysis domain, sensitivity analysis could not be conducted. All other domains were deemed to be of low risk of bias and concern for applicability. A detailed description of the risk of bias assessment is provided in Supplementary File 3.

## Discussion

This review sought to evaluate the predictive accuracy of prognostic models for Southeast Asian populations with AMI. The two validated models, namely, GRACE and TIMI scores, demonstrated good predictive accuracies in this population although some validations were underpowered. Recalibration of the GRACE score did not demonstrate a significant increase in predictive prediction. Although there are advantages to having local population-derived prediction models, the new models are presented with a longer list of predictors, limited generalizability, and marginal changes in predictive accuracy. Findings of the review were limited due to the small number and relatively higher risk of bias in the included studies.

Most predictive models are developed from specific cohorts, and therefore external validation in a different population examines the generalizability of the model. Morrow and colleagues developed the TIMI Risk Score for STEMI to predict 30-day mortality amongst STEMI patients (36). The model was derived from the Intravenous nPA for Treatment of Infarcting Myocardium Early II (InTIME II) trial and included 14,114 STEMI patients from more than 800 hospitals worldwide. The eight predictors of the TIMI Risk Score for STEMI are age, systolic blood pressure <100 mmHg, heart rate >100 bpm, Killip class II-IV, anterior ST elevation or left-bundle branch block, history of diabetes, hypertension or angina, weight <67 kg, and time to treatment >4 h. As this TIMI score is exclusively used for STEMI individuals, other variations of the TIMI risk score such as TIMI risk score for Unstable Angina/NSTEMI and Dynamic TIMI risk score exist (37, 38). Generally, external validation of these scores demonstrated good predictive accuracy in various populations (39–41).

The GRACE risk score is a scoring system to risk-stratify patients with acute coronary syndrome (ACS) to estimate their 6-month to 3-year mortality (42). Following are the eight predictors included in the original GRACE risk score: older age, history of myocardial infarction, history of heart failure, the increased pulse rate at presentation, lower systolic blood pressure at presentation, elevated initial serum creatinine level, high initial serum cardiac biomarker levels, and ST-segment elevation. In general, validation studies of the GRACE score also provided good predictive accuracies, even amongst indigenous populations, with some exceptions (43–45). Despite having good discriminative power and thus identifying higher-risk patients, there has been evidence of both overestimation and underestimation of outcomes risks of the GRACE risk score in different populations (46–50). Compared to the TIMI score, evidence shows that the GRACE risk score was more accurate in predicting long-term mortality (51). A meta-analysis also concurred that the GRACE risk score performed better than the TIMI risk score for both short- and long-term outcomes (52).

In a validation study in Japan, both original GRACE and simplified GRACE 2.0 risk scores were demonstrated to perform equally well (53), whereas some locally specific risk scores report better predictive accuracies than validation studies of the TIMI risk score and the GRACE risk scores (54, 55). This is expected as the model parameters of these risk scores are tailored to that of the development population and overfitting of the model may occur due to repeated model tuning. Characteristics of these development cohorts differ in terms of their geographic location, patient characteristics, treatment, and availability of resources to existing risk scores. However, a high predictive accuracy (high discrimination and good calibration measures) does not translate to clinical utility. Essentially, prognostic prediction scores that are simple and easy to use with accessible clinical variables promote clinical usefulness.

Patients with established CVD are at a higher risk of cardiovascular event recurrence or death, hence making secondary prevention and accurate prognosis prediction increasingly important to avert subsequent CVD events and to improve life expectancy (56). The usage of CVD risk prediction models has not only provided a more reliable outcome prediction to clinicians but also complemented decision-making to identify those at higher risk and to enhance informed decision-making with the patients while ensuring the cost-effectiveness of care (57). Usage of prognostic risk models for patients presenting with an AMI event, or any coronary artery disease event, risk stratification may be based on the timing of assessment, pre-treatment (such as TIMI score and GRACE score), post-treatment (such as CADILLAC risk score and Dynamic TIMI score), and follow-up (such as DAPT score and GUSTO score) (58).

With the abundance of existing and new prediction risk scores/models, adequate discrimination, calibration, and generalizability determine its usefulness. Overall, we observed that the external validation studies provided a rather good predictive accuracy of the models in the respective populations. However, the method of predictive accuracy measurement was insufficient in most studies thereby providing a mixed accuracy effect. The low strength of evidence in this review was influenced by the inadequate methods of predictive measurement of models, relatively higher risk of bias in almost all of the included studies, small sample size, and a small quantity of included studies. A relatively higher risk of bias in validation studies has also been reported in other reviews (59–61). Although discrimination was the most widely reported measurement, the overall accuracy of the predicted risk could not be established without its corresponding calibration measure. The C-statistic value, for example, is difficult to translate directly into clinical practice (62, 63). A very well discriminating model (C-statistic >0.8) may still be clinically irrelevant if the decision threshold is outside the range of predictions provided by the model. Additionally, the value of the C-statistic is limited in risk categorization and in balancing misclassification errors (62, 64). However, it is also important to note that calibration measures too should not be evaluated independently. Interpretation of calibration measures is influenced by arbitrary groupings of patients, poor power in small data sets, and the reporting of only *p*-value (65). Although reporting confidence intervals with the *p*-value is beneficial, this has not been widely practiced. Models demonstrating poor calibration (*p* < 0.05) generally result from statistical overfitting, measurement errors, and heterogeneity in populations (66). Heterogeneity may exist from the varying disease incidence or prevalence, patient management, and treatment policies (66). Therefore, the interpretation of predictive measures must be performed with caution. Applicability of the model to contemporary and local clinical practice as well as the additional benefit of using the model in current practice should be considered when assessing the clinical usefulness of a risk prediction model (67). Although the new models were derived from the respective local population, their applicability is also limited by having a long list of predictors. The longer list not only increases complexity and time for usage, but several of the predictors are only available in PCI-capable centers. The changes in the discriminative power in these models were also marginal and uncertainties in the improvements of classification as compared to the existing models.

Meta-analysis has the advantage of summarizing quantitative information from related studies (68). However, the heterogeneity of included studies and the type of analysis should be considered during interpretation. In this review, differences were observed in study population characteristics such as age and diagnosis, interventions received, geographical locations, and other eligibility criteria. By adopting a random effect model in the quantitative synthesis, the statistical model assumes that the underlying true parameters vary across the study populations (69). This model is beneficial as it reflects real-life differences in characteristics and treatment as well as sampling variability.

With the growing evidence of clinical prediction models, there are still limited studies performed in regions other than Europe and North America (57, 70). A review of CVD prognostic models in Latin America and the Caribbean provided evidence from only 8 studies and had similar methodological concerns (71). In another review on the diagnostic accuracy of the HEART Score to predict MACE, the Asian population was only evaluated in 4 out of 25 included studies (72). Even then, the low-risk HEART Score Asia-Pacific group reported the highest occurrence of MACE compared to other geographical locations (72). Despite the inclusion of studies from different regions, the absence of a geographical subgroup analysis prohibits a more specific evidence synthesis (73).

This study has the advantage of being the first review to systematically evaluate prognostic models for the Southeast Asian population with AMI. We performed a comprehensive search with a careful selection of studies and extensive data extraction on the key characteristics of prognostic prediction models that included information on the predictors, outcomes, and the studied population. Using the CHARMS checklist for the critical appraisal of the included studies and the PROBAST guidelines for the ROB assessment, we also have the advantage of a systematic and comprehensive review of the included studies. To ensure that the predictive measurement results are not guided by other populations, we included studies that exclusively validate or develop prediction models in the Southeast Asian population. Unfortunately, this review is limited to only patients with AMI as index disease and the inclusion of a limited number of studies and relatively higher risk of bias in included studies.

## Conclusion

Despite the wide range of prognostic models, there are still insufficient efforts to externally validate these models in the Southeast Asian population. Our evidence demonstrated a relatively good discrimination ability of both TIMI and GRACE scores, but with the limited strength of evidence, these results should be interpreted with caution. The available evidence is limited due to a small number of countries and due to the significant methodological concerns of the included studies. Future higher-quality studies spanning various parts of the Asian region will help to understand the prognostic utility of these models better.

## Data availability statement

The original contributions presented in the study are included in the article/supplementary material, further inquiries can be directed to the corresponding author.

## Author contributions

SI conceived the review, screened, and selected studies, extracted data, assessed the risk of bias, performed the statistical analysis, and wrote the review. MK screened and selected studies, extracted data, and assessed the risk of bias. MM and SA wrote the review. All authors contributed to the article and approved the submitted version.

## Conflict of interest

The authors declare that the research was conducted in the absence of any commercial or financial relationships that could be construed as a potential conflict of interest.

## Publisher's note

All claims expressed in this article are solely those of the authors and do not necessarily represent those of their affiliated organizations, or those of the publisher, the editors and the reviewers. Any product that may be evaluated in this article, or claim that may be made by its manufacturer, is not guaranteed or endorsed by the publisher.
