# Supplementary material for: Systematic review and meta-analysis of prognostic models in Southeast Asian populations with acute myocardial infarction
Source: Front Cardiovasc Med. 2022 Jul 26;9:921044. doi: 10.3389/fcvm.2022.921044 (PMC9360484; doi:10.3389/fcvm.2022.921044)
Supplement: Supplementary file 2 [file Data_Sheet_2.PDF]

Supplementary File 2

Search strategy

MEDLINE

Search ((((((((((Malaysia) OR Singapore) OR Brunei) OR Indonesia) OR Myanmar) OR Vietnam) OR Thailand) OR Cambodia) OR Laos) OR Timor-Leste)) AND (((((((((myocardial infarction) OR acute coroanry syndrome) OR ST elevation myocardial infarction) OR STEMI) OR non-ST elevation myocardial infarction) OR NSTEMI))) AND (((((((((((((((((((syntax score) OR syntax score II) OR Euroscore II) OR STS score) OR TIMI risk score) OR GRACE risk score) OR Simple risk score) OR Action-GWTG model) OR Cadillac risk score) OR PAMI risk score) OR Zwolle risk score) OR GRACE hospital discharge score) OR Dynamic TIMI risk score) OR Risk-PCI score) OR EPICOR prognostic model) OR Residual Syntax score) OR APEX-AMI risk score) OR DAPT score) OR Gusto score)) OR ((((((((predict* AND model*) OR predict* AND instrument*) OR predict* AND scor*) OR prognos* AND model*) OR prognos* AND instrument*) OR prognos* AND scor*) OR risk model*) OR risk instrument*) OR risk scor*))))

CENTRAL

#1	Malaysia OR Singapore OR Brunei OR Indonesia OR Myanmar OR Vietnam OR Thailand or Cambodia OR Laos OR Timor-Leste
#2	myocardial infarction OR acute coronary syndrome OR ST-elevation myocardial infarction OR STEMI OR NSTEMI OR non-ST elevation myocardial infarction
#3	MeSH descriptor: [Coronary Artery Disease] explode all trees
#4	MeSH descriptor: [Myocardial Infarction] explode all trees
#5	#2 OR #3 OR #4
#6	prediction model OR prediction instrument OR prediction score OR prognostic model OR prognostic instrument OR prognostic score OR risk model OR risk risk instrument OR risk score
#7	Syntax score OR Syntax score II OR Euroscore II OR STS score OR TIMI risk score OR GRACE score OR Simple risk score OR ACtion-GQTG model OR Cadillac risk score OR PAMI risk score OR Zwolle risk score OR Grace hospital discharge score OR Dynamic TIMI risk score OR Risk-PCI score OR EPICOR prognostic model OR APEX-AMI risk score OR Residual Syntax score OR DAPT score OR Gusto score
#8	#6 OR #7
#9	#1 AND #5 AND #8
