# Supplementary material for: Systematic review and meta-analysis of prognostic models in Southeast Asian populations with acute myocardial infarction
Source: Front Cardiovasc Med. 2022 Jul 26;9:921044. doi: 10.3389/fcvm.2022.921044 (PMC9360484; doi:10.3389/fcvm.2022.921044)
Supplement: Supplementary file 3 [file Data_Sheet_3.PDF]

Supplementary File 3

Risk of Bias Assessment Using PROBAST

1 AZIZ 2021

Classify the evaluation based on its aim			
**Type of prediction study**	**PROBAST boxes to complete**	**Tick as appropriate**	**Definition for type of prediction model study**
Development only	Development	✓	Prediction model development without external validation. These studies may include internal validation methods, such as bootstrapping and cross-validation techniques.
Development and validation	Development and validation		Prediction model development combined with external validation in other participants in the same article.
Validation only	Validation	✓	External validation of existing (previously developed) model in other participants.

**Publication reference**	Aziz F, Malek S, Ibrahim KS, Raja Shariff RE, Wan Ahmad WA, Ali RM, et al. Short- and long-term mortality prediction after an acute ST-elevation myocardial infarction (STEMI) in Asians: A machine learning approach. PLoS One. 2021;16(8):e0254894
**Models of interest**	TIMI score and SVMvarImp-SBE-SVM
**Outcome of interest**	In-hospital mortality, 30-day mortality, and 1-year mortality

**DOMAIN 1: Participants**			
**A. Risk of Bias**			
Describe the sources of data and criteria for participant selection:<br>"We used retrospective data from the Malaysian National Cardiovascular Database (NCVD-ACS) registry collected between 2006 until 2016. The NCVD registry was approved by the Medical Review & Ethics Committee (MREC), Ministry of Health (MOH) Malaysia in 2007 (Approval Code: NMRR-07-20-250). MREC waived informed patient consent for NCVD. The registry collects data on a standardised set of clinical, demographic, and procedural variables, along with outcomes, for consecutive patients treated at participating institutions"			
		Dev	Val
1.1 Were appropriate data sources used, e.g. cohort, RCT or nested case-control study data?			**N**
1.2 Were all inclusions and exclusions of participants appropriate?			**Y**
**Risk of bias introduced by selection of participants**	**RISK:**		**HIGH**
Rationale of bias rating:<br>Participant data were obtained from an existing database			
**B. Applicability**			
Describe included participants, setting and dates:<br>"We used retrospective data from the Malaysian National Cardiovascular Database (NCVD-ACS) registry collected between 2006 until 2016 "			
**Concern that the included participants and setting do not match the review question**	**CONCERN:**		**LOW**
Rationale of applicability rating:<br>Included participants and setting were reasonable for the review question.			

**DOMAIN 2: Predictors**
**A. Risk of Bias**
List and describe predictors included in the final model, e.g. definition and timing of assessment:<br>**SVMvarImp-SBE-SVM**<br>- *In-hospital mortality:* Age, family history of premature CVD, chronic renal disease, heart rate, systolic BP, diastolic BP, Killip class, fasting blood glucose, ECG-type bundle branch block, ECG- location lateral lead, cardiac catheterization, Beta blockers, statin, diuretics, and oral hypoglycaemic agent.

-30-day mortality: Age, race, family history of premature CVD, heart rate, Killip class, HDL, fasting blood glucose, cardiac catheterization, PCI, ASA, Beta blockers, diuretics, and insulin.<br>- *1-year mortality:* Age, smoking status, hypertension, diabetes, heart rate, systolic BP, Killip class, fasting blood glucose, PCI, ACE inhibitor, diuretics, and insulin.			
		Dev	Val
2.1 Were predictors defined and assessed in a similar way for all participants?			PY
2.2 Were predictor assessments made without knowledge of outcome data?			PY
2.3 Are all predictors available at the time the model is intended to be used?			Y
**Risk of bias introduced by predictors or their assessment**	**RISK:**		**LOW**
Rationale of bias rating:<br>In view of the usage of a registry database for the participant predictors assessment, we believe that there is low risk of bias in this domain.			
**B. Applicability**			
Concern that the definition, assessment, or timing of predictors in the model do not match the review question	**CONCERN:**		**LOW**
Rationale of applicability rating:<br>Definition, assessment, and timing of predictors in the model seem reasonable and acceptable to the review question.			

**DOMAIN 3: Outcome**			
**A. Risk of Bias**			
Describe the outcome, how it was defined and determined, and the time interval between predictor assessment and outcome determination:<br>*"*The mortality time frame was calculated from first hospital admission for in-hospital, 30 days and 1-year. Confirmation of deaths was done yearly via record linkages with the Malaysian National Registration Department"			
		Dev	Val
3.1 Was the outcome determined appropriately?			PY
3.2 Was a pre-specified or standard outcome definition used?			Y
3.3 Were predictors excluded from the outcome definition?			Y
3.4 Was the outcome defined and determined in a similar way for all participants?			PY
3.5 Was the outcome determined without knowledge of predictor information?			PY
3.6 Was the time interval between predictor assessment and outcome determination appropriate?			Y
**Risk of bias introduced by the outcome or its determination**	**RISK:**		**LOW**
Rationale of bias rating:<br>The description and determination of outcomes are deemed reasonable and acceptable.			
**B. Applicability**			
At what time point was the outcome determined:<br>"The mortality time frame was calculated from first hospital admission for in-hospital, 30 days and 1-year."			
**Concern that the outcome, its definition, timing or determination do not match the review question**	**CONCERN:**		**LOW**
Rationale of applicability rating:<br>The outcome, its definition, timing, and determination seem reasonable and acceptable to the review question.			

**DOMAIN 4: Analysis**
**Risk of Bias**
Describe numbers of participants, number of candidate predictors, outcome events and events per candidate predictor:<br>Number of participants: 6,299 for In-hospital, 3130 for 30-days, and 2939 for 1-year<br>Outcome events: 338 for In-hospital, 252 for 30-days, and 423 for 1-year<br><br>**TIMI Score**<br>Number of candidate predictors: 9<br>Events per candidate predictor: 38 for In-hospital, 28 for 30-days, and 47 for 1-year<br>**SVMvarImp-SBE-SVM**<br>Number of candidate predictors: 15 for In-hospital, 13 for 30-days, and 12 for 1-year<br>Events per candidate predictor: 23 for In-hospital, 19 for 30-days, and 35 for 1-year
Describe how the model was developed:

TIMI Score *– Not applicable*

SVMvarImp-SBE-SVM – "Prediction models post-STEMI were developed using three selected ML algorithms. Next, feature selection was carried out on the ranked variables in an ascending order iteratively. 10-fold cross-validation was used to avoid overfitting for model development on the training set. The prediction models were trained and tested for each iteration, and the models with the highest performance consisting of the least number of variables were selected. Predictive performances of the models were calculated using the validation dataset."

Describe whether and how the model was validated, either internally (e.g. bootstrapping, cross validation, random split sample) or externally (e.g. temporal validation, geographical validation, different setting, different type of participants):

TIMI Score – Temporal and geographical validation

SVMvarImp-SBE-SVM – "Data were split for model development (70%) and validation (30%)."\

Describe the performance measures of the model, e.g. (re)calibration, discrimination, (re)classification, net benefit, and whether they were adjusted for optimism:

TIMI Score

**Outcome**	**Calibration measure**	**Discrimination measure**
In-hospital mortality	Not reported	0.81 (0.77 – 0.80)
30-day mortality	Not reported	0.92 (0.88 – 0.95)
1-year mortality	Not reported	0.80 (0.75 – 0.84)

SVMvarImp-SBE-SVM

**Outcome**	**Calibration measure**	**Discrimination measure**	**Nett Reclassification Index**
In-hospital mortality	Not reported	0.88 (0.85 – 0.91)	0.20 (p<0.0001)
30-day mortality	Not reported	0.90 (0.87 – 0.94)	0.19 (p<0.0001)
1-year mortality	Not reported	0.84 (0.80 – 0.87)	0.14 (p<0.0001)

Describe any participants who were excluded from the analysis:

Not reported

Describe missing data on predictors and outcomes as well as methods used for missing data:

"We developed the ML using a complete set of data to ensure the validity of the findings. A total of 27,592 STEMI cases from the registry were collected 12,368 were identified as complete cases (with no missing values on predictors). Out of the 12 368 datasets, a total of 6299, 3130 and 2939 complete cases were used for in-hospital, 30-days and 1-year respectively for the model development. This rendered almost 50% complete cases of patients with a full predictor set of 50 variables for each time frame (9 continuous, 41 categorical) for the study"

"Secondary analyses were carried out after adding 15224 missing cases imputed using multi- variable imputation using chained equations and predictive mean matching that yields a total of 27 592 cases. This method imputes missing values based on real values from other cases where predicted values are closest. Our reference for incomplete dataset refers to missing sets of variables up to 50%. The missing dataset mentioned refers to patient characteristics and not outcome data. As our dataset is a prospective dataset, with retrospective data management, the level of missingness in values across all variables was completely random and beyond our control. The probability of missingness in our dataset depends neither on the observed values in any variable of the dataset nor on the unobserved part of the dataset.

Hence the dataset is classified as missing completely at random (MCAR) which is the highest level of randomness and it implies that the pattern of missing value is random and does not depend on any variable which may or may not be included in the analysis. We had complete data for all our outcomes. The models were tested with a similar validation dataset for ML models trained with a complete cases dataset. "

	Dev	Val
4.1 Were there a reasonable number of participants with the outcome?		Y
4.2 Were continuous and categorical predictors handled appropriately?		Y
4.3 Were all enrolled participants included in the analysis?		Y
4.4 Were participants with missing data handled appropriately?		Y
4.5 Was selection of predictors based on univariable analysis avoided?		
4.6 Were complexities in the data (e.g. censoring, competing risks, sampling of controls) accounted for appropriately?		Y
4.7 Were relevant model performance measures evaluated appropriately?		Y
4.8 Were model overfitting and optimism in model performance accounted for?		

4.9 Do predictors and their assigned weights in the final model correspond to the results from multivariable analysis?		NI	
**Risk of bias introduced by the analysis**	**RISK:**		**LOW**
Rationale of bias rating: The description of analysis and its results are deemed reasonable and acceptable.			

**Overall judgement about risk of bias and applicability of the prediction model evaluation**		
**Overall judgement of risk of bias**	**RISK:**	**HIGH**
Summary of sources of potential bias: Overall potential source of bias is judged as high risk due to concerns in the participants domain		
**Overall judgement of applicability**	**CONCERN:**	**LOW**
Summary of applicability concerns: The overall applicability of the study participants, predictors and outcomes were of all low concern.		

2 Bulluck 2019

**Classify the evaluation based on its aim**			
**Type of prediction study**	**PROBAST boxes to complete**	**Tick as appropriate**	**Definition for type of prediction model study**
Development only	Development		Prediction model development without external validation. These studies may include internal validation methods, such as bootstrapping and cross-validation techniques.
Development and validation	Development and validation	✓	Prediction model development combined with external validation in other participants in the same article.
Validation only	Validation		External validation of existing (previously developed) model in other participants.

**Publication reference**	Bulluck H, Zheng H, Chan MY, Foin N, Foo DC, Lee CW, et al. Independent Predictors of Cardiac Mortality and Hospitalization for Heart Failure in a Multi-Ethnic Asian ST-segment Elevation Myocardial Infarction Population Treated by Primary Percutaneous Coronary Intervention. Sci Rep. 2019;9(1):10072.
**Models of interest**	Singapore Myocardial Infarction Registry (SMIR)
**Outcome of interest**	In-hospital cardiac mortality, 30-day cardiac mortality, 1-year cardiac mortality and 1-year hospitalisation for heart failure

**DOMAIN 1: Participants**			
**A. Risk of Bias**			
Describe the sources of data and criteria for participant selection: “This was a retrospective study on data collected prospectively between 2008 and 2015 by the National Registry of Diseases Office in SMIR. Data collection on all AMI cases from all public and private hospitals is mandated and funded by the state and yearly reports are generated for the Ministry of Health.” “The main inclusion criteria were patients presenting to hospital with a STEMI within 12 hours of symptoms onset and were reperfused by PPCI. Patients with a STEMI but not reperfused by PPCI or those with a LBBB were excluded “			
		Dev	Val
1.3 Were appropriate data sources used, e.g. cohort, RCT or nested case-control study data?		N	**N**
1.4 Were all inclusions and exclusions of participants appropriate?		Y	**Y**
**Risk of bias introduced by selection of participants**	**RISK:**	**High**	**High**
Rationale of bias rating: Inclusion criteria and method of recruitment seem reasonable and appropriate, but the study has used registry data for the prognostic model			
**B. Applicability**			
Describe included participants, setting and dates: “Data collected prospectively between 2008 and 2015 by the National Registry of Diseases Office in SMIR”			
**Concern that the included participants and setting do not match the review question**	**CONCERN:**	**LOW**	**LOW**
Rationale of applicability rating: Description of included participants, setting and dates seem reasonable to the review question.			
**DOMAIN 2: Predictors**			
**A. Risk of Bias**			

4

List and describe predictors included in the final model, e.g. definition and timing of assessment:<br>*Predictors:* Age, history of diabetes, history of ischaemic heart disease, Killip class, Creatinine, Haemoglobin, Troponin T/I, Ischaemic Time, and LVEF during hospitalisation.<br>"The main outcomes of interest were in-hospital cardiac mortality, 30-day cardiac mortality, 1-year cardiac mortality and 1-year HHF."

		Dev	Val
2.4 Were predictors defined and assessed in a similar way for all participants?		**Y**	**Y**
2.5 Were predictor assessments made without knowledge of outcome data?		**PY**	**PY**
2.6 Are all predictors available at the time the model is intended to be used?		**Y**	**Y**
**Risk of bias introduced by predictors or their assessment**	**RISK:**	**LOW**	**LOW**
Rationale of bias rating:<br>Predictors used in this study together with their assessment seem reasonable and low risk of bias.			
**B. Applicability**			
Concern that the definition, assessment, or timing of predictors in the model do not match the review question	**CONCERN:**	**LOW**	**LOW**
Rationale of applicability rating:<br>The definition, assessment, and timing of predictors in the model seem reasonable to the review question.			

**DOMAIN 3: Outcome**			
**A. Risk of Bias**			
Describe the outcome, how it was defined and determined, and the time interval between predictor assessment and outcome determination:<br>"The main outcomes of interest were in-hospital cardiac mortality, 30-day cardiac mortality, 1-year cardiac mortality and 1-year HHF. Specifically for HHF, the data was available for 2008 to 2013 only. Patients who died during hospitalization for STEMI were excluded for this outcome. Among patients discharged alive, patients who died due to HF within 1 year from STEMI discharge but without HF admission were included. However, patients who died due to non-HF causes within 1 year from STEMI discharge and without HF admission were excluded."<br>*"*Cardiac mortality was identified as death occurring from a cardiac cause and defined by the ICD codes (ICD-9: 391–398, 402, 410–429; ICD-10: I00-I52 except I26). The time of death was extracted from death certificates obtained from the Registry of Births and Deaths. "			
		Dev	Val
3.7 Was the outcome determined appropriately?		**Y**	**Y**
3.8 Was a pre-specified or standard outcome definition used?		**Y**	**Y**
3.9 Were predictors excluded from the outcome definition?		**PY**	**PY**
3.10 Was the outcome defined and determined in a similar way for all participants?		**Y**	**Y**
3.11 Was the outcome determined without knowledge of predictor information?		**PY**	**PY**
3.12 Was the time interval between predictor assessment and outcome determination appropriate?		**Y**	**Y**
**Risk of bias introduced by the outcome or its determination**	**RISK:**	**LOW**	**LOW**
Rationale of bias rating:<br>The description and determination of outcomes are deemed reasonable and acceptable.			
**B. Applicability**			
At what time point was the outcome determined:<br>"The main outcomes of interest were in-hospital cardiac mortality, 30-day cardiac mortality, 1-year cardiac mortality and 1-year HHF."			
**Concern that the outcome, its definition, timing, or determination do not match the review question**	**CONCERN:**	**LOW**	**LOW**
Rationale of applicability rating:<br>The outcome, its definition, timing, and determination seem reasonable and acceptable to the review question.			

**DOMAIN 4: Analysis**
**Risk of Bias**
Describe numbers of participants, number of candidate predictors, outcome events and events per candidate predictor:<br>Number of participants: *11,546*<br>Outcome events: 741 for in-hospital mortality, 780 for 30-day cardiac mortality, 956 for 1-year mortality, and 399 for 1-year hospitalisation for heart failure<br>Number of candidate predictors: 10

Events per candidate predictor: 74 for in-hospital mortality, 78 for 30-day cardiac mortality, 96 for 1-year mortality, and 40 for 1-year hospitalisation for heart failure
Describe how the model was developed:<br>Multivariable logistic regression
Describe whether and how the model was validated, either internally<br>Internal validity was assessed using bootstrapping techniques on the derivation cohort. External validity was assessed using the validation cohort.
Describe the performance measures of the model:

	Calibration	Discrimination (C-statistics (95%CI)	Misclassification rate
In-hospital cardiac mortality	Not reported	0.922 (0.902 -0.942)	14%
30-day cardiac mortality	Not reported	0.913 (0.891 – 0.935)	14.7%
1-year cardiac mortality	Not reported	0.903 (0.882 – 0.923)	16.2%
1-year HHF	Not reported	0.855 (0.823 – 0.888)	24%

		Dev	Val
Describe any participants who were excluded from the analysis:<br>Missing data were handled as case deletion without any imputation.			
Describe missing data on predictors and outcomes as well as methods used for missing data:<br>No patient was lost to follow-up for all mortality outcomes.			
4.10 Were there a reasonable number of participants with the outcome?		Y	Y
4.11 Were continuous and categorical predictors handled appropriately?		Y	Y
4.12 Were all enrolled participants included in the analysis?		Y	Y
4.13 Were participants with missing data handled appropriately?		N	N
4.14 Was selection of predictors based on univariable analysis avoided?		Y	
4.15 Were complexities in the data (e.g. censoring, competing risks, sampling of controls) accounted for appropriately?		NI	NI
4.16 Were relevant model performance measures evaluated appropriately?		Y	Y
4.17 Were model overfitting and optimism in model performance accounted for?		NI	
4.18 Do predictors and their assigned weights in the final model correspond to the results from multivariable analysis?		NI	
**Risk of bias introduced by the analysis**	**RISK:**	**HIGH**	**HIGH**
Rationale of bias rating:<br>The description of analysis and its results are deemed high risk due to exclusion of participants with missing data.			

**Overall judgement about risk of bias and applicability of the prediction model evaluation**		
**Overall judgement of risk of bias**	**RISK:**	**HIGH**
Summary of sources of potential bias:<br>The sources of potential bias for two domains were at low risk except for the participants and analysis domains.		
**Overall judgement of applicability**	**CONCERN:**	**LOW**
Summary of applicability concerns:<br>The overall applicability of the study participants, predictors and outcomes were of all low concern.		

3 Chan 2011

**Classify the evaluation based on its aim**			
**Type of prediction study**	**PROBAST boxes to complete**	**Tick as appropriate**	**Definition for type of prediction model study**
Development only	Development		Prediction model development without external validation. These studies may include internal validation methods, such as bootstrapping and cross-validation techniques.
Development and validation	Development and validation	✓	Prediction model development combined with external validation in other participants in the same article.
Validation only	Validation		External validation of existing (previously developed) model in other participants.

**Publication reference**	Chan MY, Shah BR, Gao F, Sim LL, Chua T, Tan HC, et al. Recalibration of the Global Registry of Acute Coronary Events risk score in a multi ethnic Asian population. Am Heart J. 2011;162(2):291-9.
**Models of interest**	GRACE Risk Score, Singapore score, Recalibrated GRACE risk score
**Outcome of interest**	All-cause mortality

**DOMAIN 1: Participants**			
**A. Risk of Bias**			
Describe the sources of data and criteria for participant selection:<br>"Patients from the Singapore Myocardial Infarction Registry, a cohort of patients with AMI admitted to 6 public hospitals in Singapore, from January 1, 2000, through December 3, 2005. Patients with AMI were identified for inclusion in the Singapore cohort using World Health Organization Multinational MONItoring of trends and determinants in CArdiovascular disease (MONICA) criteria at admission, discharge International Classification of Diseases, Ninth Revision codes 410.00 to 414.19, or post-mortem reports. Patients younger than 21 years, who were non-residents, and, as in the original GRACE cohort, who died within 24 hours of admission were excluded from the current analysis"			
		Dev	Val
1.5 Were appropriate data sources used, e.g. cohort, RCT or nested case-control study data?		Y	**Y**
1.6 Were all inclusions and exclusions of participants appropriate?		Y	**Y**
**Risk of bias introduced by selection of participants**	**RISK:**	**LOW**	**LOW**
Rationale of bias rating:<br>Inclusion criteria and method of recruitment seem reasonable and appropriate			
**B. Applicability**			
Describe included participants, setting and dates:<br>"Patients from the Singapore Myocardial Infarction Registry, a cohort of patients with AMI admitted to 6 public hospitals in Singapore, from January 1, 2000, through December 3, 2005".			
**Concern that the included participants and setting do not match the review question**	**CONCERN:**	**LOW**	**LOW**
Rationale of applicability rating:<br>Description of included participants, setting and dates seem reasonable to the review question.			

**DOMAIN 2: Predictors**			
**A. Risk of Bias**			
List and describe predictors included in the final model, e.g. definition and timing of assessment:<br>"To validate the GRACE risk score in the Singapore cohort, we examined the 8 risk factors reported to be the strongest predictors of mortality: age, initial serum creatinine, systolic blood pressure, heart rate, initial cardiac biomarker elevation, Killip class, ST-segment deviation, and cardiac arrest at presentation".			
		Dev	Val
2.7 Were predictors defined and assessed in a similar way for all participants?		**Y**	**Y**
2.8 Were predictor assessments made without knowledge of outcome data?		**PY**	**PY**
2.9 Are all predictors available at the time the model is intended to be used?		**Y**	**Y**
**Risk of bias introduced by predictors or their assessment**	**RISK:**	**LOW**	**LOW**
Rationale of bias rating:<br>Predictors used in this study together with their assessment seem reasonable and low risk of bias.			
**B. Applicability**			
Concern that the definition, assessment, or timing of predictors in the model do not match the review question	**CONCERN:**	**LOW**	**LOW**
Rationale of applicability rating:<br>The definition, assessment, and timing of predictors in the model seem reasonable to the review question.			

**DOMAIN 3: Outcome**		
**A. Risk of Bias**		
Describe the outcome, how it was defined and determined, and the time interval between predictor assessment and outcome determination:<br>*"W*e examined the 8 risk factors reported to be the strongest predictors of mortality: age, initial serum creatinine, systolic blood pressure, heart rate, initial cardiac biomarker elevation, Killip class, ST- segment deviation, and cardiac arrest at presentation. The outcome measure for the GRACE risk score was all-cause mortality during index hospitalization for AMI".		
	Dev	Val

3.13 Was the outcome determined appropriately?		**Y**	**Y**
3.14 Was a pre-specified or standard outcome definition used?		**Y**	**Y**
3.15 Were predictors excluded from the outcome definition?		**PY**	**PY**
3.16 Was the outcome defined and determined in a similar way for all participants?		**Y**	**Y**
3.17 Was the outcome determined without knowledge of predictor information?		**N**	**N**
3.18 Was the time interval between predictor assessment and outcome determination appropriate?		**Y**	**Y**
**Risk of bias introduced by the outcome or its determination**	**RISK:**	**LOW**	**LOW**

Rationale of bias rating:
As this study extracted registry data, the determination of outcome is at low risk of bias.

B. Applicability

At what time point was the outcome determined:
"The outcome measure for the GRACE risk score was all-cause mortality during index hospitalization for AMI".

**Concern that the outcome, its definition, timing or determination do not match the review question**	**CONCERN:**	**LOW**	**LOW**

Rationale of applicability rating:
The outcome, its definition, timing and its determination seem reasonable to the review question.

DOMAIN 4: Analysis

Risk of Bias

Describe numbers of participants, number of candidate predictors, outcome events and events per candidate predictor:
15,151 study participants. Number of events by race: Chinese 894 (9.78%); Malay 207 (7.63%); Indian 120 (6.43%).

Describe how the model was developed
Logistic regression

Describe whether and how the model was validated, either internally
GRACE Risk score was validated geographically and temporally, while the newly derived Singapore score and recalibrated GRACE Risk Score were not.

Describe the performance measures of the model, e.g. (re)calibration, discrimination, (re)classification, net benefit, and whether they were adjusted for optimism:

	Calibration results	*Discrimination results*	*Classification results*
External validation of GRACE Risk Score	Hosmer-Lemeshow: Chinese 291.77 (<0.001); Malay 55.77 (p <0.001); Indian 39.69 (p <0.001)	C- statistic: Chinese 0.86 (95%CI: 0.85-0.88); Malay 0.86 (95%CI: 0.84-0.89); Indian 0.84 (95%CI: 0.81-0.88)	Not reported
Singapore Score	Hosmer-Lemeshow: Chinese 24.33 (p= 0.002); Malay 7.21 (p= 0.514); Indian 6.55 (p = 0.586)	C- statistic: Chinese 0.88 (95%CI: 0.87-0.90); Malay 0.89 (95%CI: 0.87-0.91); Indian 0.88 (95%CI: 0.84-0.91)	Not reported
Recalibrated GRACE Risk Score	Hosmer-Lemeshow: Chinese 89.66 (p= 0.001); Malay 24.33 (p =0.002); Indian 18.32 (p = 0.019)	C- statistic: Chinese 0.86 (95%CI: 0.85-0.88); Malay 0.86 (95%CI: 0.84-0.89); Indian 0.84 (95%CI: 0.81-0.88)	Not reported

Describe any participants who were excluded from the analysis:
"Data were missing for 1 or more of the 8 GRACE risk factors in 9.53% of Chinese, 9.68% of Malay, and 8.80% of Indian subjects. No imputation of data was performed, and subjects with missing data were excluded in the model validation analysis".

Describe missing data on predictors and outcomes as well as methods used for missing data:
"Data were missing for 1 or more of the 8 GRACE risk factors in 9.53% of Chinese, 9.68% of Malay, and 8.80% of Indian subjects. No imputation of data was performed, and subjects with missing data were excluded in the model validation analysis".

	Dev	Val
4.19 Were there a reasonable number of participants with the outcome?	Y	Y
4.20 Were continuous and categorical predictors handled appropriately?	Y	Y
4.21 Were all enrolled participants included in the analysis?	N	N
4.22 Were participants with missing data handled appropriately?	N	N
4.23 Was selection of predictors based on univariable analysis avoided?	Y	

8

4.24 Were complexities in the data (e.g. censoring, competing risks, sampling of controls) accounted for appropriately?		NI	NI
4.25 Were relevant model performance measures evaluated appropriately?		Y	Y
4.26 Were model overfitting and optimism in model performance accounted for?		NI	
4.27 Do predictors and their assigned weights in the final model correspond to the results from multivariable analysis?		Y	
**Risk of bias introduced by the analysis**	**RISK:**	**HIGH**	**HIGH**
Rationale of bias rating: Participants with missing data were excluded from the analysis.			

**Overall judgement about risk of bias and applicability of the prediction model evaluation**		
**Overall judgement of risk of bias**	**RISK:**	**HIGH**
Summary of sources of potential bias: The sources of potential bias for three domains were at low risk except for analysis domain.		
**Overall judgement of applicability**	**CONCERN:**	**LOW**
Summary of applicability concerns: The overall applicability of the study participants, predictors and outcomes were of all low concern.		

4 Chotechuang 2016

**Classify the evaluation based on its aim**			
**Type of prediction study**	**PROBAST boxes to complete**	**Tick as appropriate**	**Definition for type of prediction model study**
Development only	Development		Prediction model development without external validation. These studies may include internal validation methods, such as bootstrapping and cross-validation techniques.
Development and validation	Development and validation		Prediction model development combined with external validation in other participants in the same article.
Validation only	Validation	✔	External validation of existing (previously developed) model in other participants.

**Publication reference**	Chotechuang Y, Phrommintikul A, Muenpa R, Patumanond J, Chaichuen T, Kuanprasert S, et al. The prognostic utility of GRACE risk score in predictive cardiovascular event rate in STEMI patients with successful fibrinolysis and delay intervention in non PCI-capable hospital: a retrospective cohort study. BMC Cardiovasc Disord. 2016;16(1):212.
**Models of interest**	GRACE score
**Outcome of interest**	30-day and 6-month composite cardiovascular outcome (death, re-hospitalisation with acute coronary syndrome, re-hospitalisation with heart failure, and stroke)

**DOMAIN 1: Participants**			
**A. Risk of Bias**			
Describe the sources of data and criteria for participant selection: "We retrospectively analysed the data from the Maharaj Nakorn Chiang Mai Hospital STEMI registry during the period 2007–2012. The STEMI patients who had successfully fibrinolysis in non PCI-capable hospital and received delayed coronary intervention (during 24 h to 14 days after successful fibrinolytic therapy) at Maharaj Nakorn Chiang Mai hospital were included for analysis in the study. The exclusion criteria were the patients who unsuccessfully fibrinolysis, received early coronary intervention (<24 h after fibrinolytic therapy), received very delayed coronary intervention (>2 weeks after fibrinolytic therapy), the patients who denied for further interventions after fibrinolysis, the patients who received primary PCI or rescue PCI and the patients who had the previous history of coronary-artery bypass surgery."			
		Dev	Val
1.7 Were appropriate data sources used, e.g. cohort, RCT or nested case-control study data?			**N**
1.8 Were all inclusions and exclusions of participants appropriate?			**Y**
**Risk of bias introduced by selection of participants**	**RISK:**		**High**
Rationale of bias rating: Inclusion criteria and method of recruitment seem reasonable and appropriate, but the study has used registry data for the prognostic model			
**B. Applicability**			

Describe included participants, setting and dates:<br>"We retrospectively analysed the data from the Maharaj Nakorn Chiang Mai Hospital STEMI registry during the period 2007–2012."			
**Concern that the included participants and setting do not match the review question**	**CONCERN:**	**LOW**	**LOW**
Rationale of applicability rating:<br>Description of included participants, setting and dates seem reasonable to the review question.			
**DOMAIN 2: Predictors**			
**A. Risk of Bias**			
List and describe predictors included in the final model, e.g. definition and timing of assessment:			
		Dev	Val
2.10 Were predictors defined and assessed in a similar way for all participants?			**Y**
2.11 Were predictor assessments made without knowledge of outcome data?			**PY**
2.12 Are all predictors available at the time the model is intended to be used?			**Y**
**Risk of bias introduced by predictors or their assessment**	**RISK:**		**LOW**
Rationale of bias rating:<br>Predictors used in this study together with their assessment seem reasonable and low risk of bias.			
**B. Applicability**			
Concern that the definition, assessment or timing of predictors in the model do not match the review question	**CONCERN:**		**LOW**
Rationale of applicability rating:<br>The definition, assessment and timing of predictors in the model seem reasonable to the review question.			

**DOMAIN 3: Outcome**			
**A. Risk of Bias**			
Describe the outcome, how it was defined and determined, and the time interval between predictor assessment and outcome determination:<br>"The primary end point for this analysis was composite outcomes, which included all-cause mortality, re-hospitalization with ACS, re-hospitalization with heart failure (HF) and stroke at 1 and 6-month. Re-hospitalized with ACS was defined as re-admission after discharge from hospital with ACS composed with clinical chest pain, rising of cardiac enzymes and dynamic ST-segment change. Re-hospitalized with heart failure was defined as re-- admission after discharge from hospital with clinical de- compensated heart failure or received intravenous diuretic. Culprit vessel PCI was defined as PCI confined to culprit vessel lesion only. The multivessel PCI was defined as PCI in which lesions in the culprit vessel as well as ≥1 non-culprit vessel lesions. "			
		Dev	Val
3.19 Was the outcome determined appropriately?			**Y**
3.20 Was a pre-specified or standard outcome definition used?			**Y**
3.21 Were predictors excluded from the outcome definition?			**PY**
3.22 Was the outcome defined and determined in a similar way for all participants?			**Y**
3.23 Was the outcome determined without knowledge of predictor information?			**PY**
3.24 Was the time interval between predictor assessment and outcome determination appropriate?			**Y**
**Risk of bias introduced by the outcome or its determination**	**RISK:**		**LOW**
Rationale of bias rating:<br>As this study extracted registry data, the determination of outcome is at low risk of bias.			
**B. Applicability**			
At what time point was the outcome determined:<br>"The primary end point for this analysis was composite outcomes, which included all-cause mortality, re-hospitalization with ACS, re-hospitalization with heart failure (HF) and stroke at 1 and 6-month."			
**Concern that the outcome, its definition, timing, or determination do not match the review question**	**CONCERN:**		**LOW**
Rationale of applicability rating:<br>The outcome, its definition, timing and its determination seem reasonable to the review question.			
**DOMAIN 4: Analysis**			
**Risk of Bias**			
Describe numbers of participants, number of candidate predictors, outcome events and events per candidate predictor:			

10

Number of participants: 88 for Low GRACE group and 64 for intermediate to high GRACE group<br>Outcome events: 2 for composite outcomes for low GRACE group and 10 for intermediate to high GRACE group at 1-month; 6 for composite outcomes for low GRACE group and 12 for intermediate to high GRACE group at 6-month<br>Number of candidate predictors: 8<br>Events per candidate predictor: 0.25 for composite outcomes for low GRACE group and 1.25 for intermediate to high GRACE group at 1-month; 0.75 for composite outcomes for low GRACE group and 1.5 for intermediate to high GRACE group at 6-month
Describe how the model was developed:<br>Not applicable
Describe whether and how the model was validated<br>Not applicable
Describe the performance measures of the model, e.g. (re)calibration, discrimination, (re)classification, net benefit, and whether they were adjusted for optimism:<br>"The prognostic utility of GRACE score on clinical outcomes was analyzed by logistic regression analysis and presented as odd ratio and area under the ROC curve (AuROC). "
Describe any participants who were excluded from the analysis:<br>Not reported
Describe missing data on predictors and outcomes as well as methods used for missing data:<br>Not reported

		Dev	Val
4.28 Were there a reasonable number of participants with the outcome?			N
4.29 Were continuous and categorical predictors handled appropriately?			Y
4.30 Were all enrolled participants included in the analysis?			PY
4.31 Were participants with missing data handled appropriately?			NI
4.32 Was selection of predictors based on univariable analysis avoided?			
4.33 Were complexities in the data (e.g. censoring, competing risks, sampling of controls) accounted for appropriately?			NI
4.34 Were relevant model performance measures evaluated appropriately?			Y
4.35 Were model overfitting and optimism in model performance accounted for?			
4.36 Do predictors and their assigned weights in the final model correspond to the results from multivariable analysis?			
**Risk of bias introduced by the analysis**	**RISK:**		**HIGH**
Rationale of bias rating:<br>There was inadequate information on handling of missing data. Additionally, the number of events per predictor is low.			

**Overall judgement about risk of bias and applicability of the prediction model evaluation**		
**Overall judgement of risk of bias**	**RISK:**	**HIGH**
Summary of sources of potential bias:<br>The sources of potential bias for two domains were at low risk except for the participants and analysis domains.		
**Overall judgement of applicability**	**CONCERN:**	**LOW**
Summary of applicability concerns:<br>The overall applicability of the study participants, predictors and outcomes were of all low concern.		

5 Chotechuang 2020

**Classify the evaluation based on its aim**			
**Type of prediction study**	**PROBAST boxes to complete**	**Tick as appropriate**	**Definition for type of prediction model study**
Development only	Development		Prediction model development without external validation. These studies may include internal validation methods, such as bootstrapping and cross-validation techniques.
Development and validation	Development and validation		Prediction model development combined with external validation in other participants in the same article.
Validation only	Validation	✓	External validation of existing (previously developed) model in other participants.

11

Publication reference	Chotechuang Y, Phrommintikul A, Kuanprasert S, Muenpa R, Ruengorn C, Patumanond J, et al. GRACE score and cardiovascular outcomes prediction among the delayed coronary intervention after post-fibrinolytic STEMI patients in a limited PCI-capable hospital. Open Heart. 2020;7(1):e001133.
**Models of interest**	GRACE score
**Outcome of interest**	30-day and 6-month composite cardiovascular outcome (death, re-hospitalisation with acute coronary syndrome, re-hospitalisation with heart failure, and stroke)

**DOMAIN 1: Participants**			
**A. Risk of Bias**			
Describe the sources of data and criteria for participant selection: "It was a retrospective cohort study. The data from Maharaj Nakorn Chiang Mai Hospital (PCI-capable hospital) STEMI registry during the period 2007–2012 were analysed. The post-fibrinolytic therapy STEMI patients who underwent a delayed coronary intervention (24 hours to 2 weeks) were included in our study. The exclusion criteria included the patients who failed fibrinolytic therapy (decreased in elevation of ST-segment <50% at 90min), performed an early coronary intervention (<24hours), underwent very long delayed coronary intervention (>2 weeks), the patients who refused for further interventions after fibrinolytic therapy, under- went PPCI or rescue PCI and previous history of coronary- artery bypass surgery. "			
		Dev	Val
1.9 Were appropriate data sources used, e.g. cohort, RCT or nested case-control study data?			**N**
1.10 Were all inclusions and exclusions of participants appropriate?			**Y**
**Risk of bias introduced by selection of participants**	**RISK:**		**High**
Rationale of bias rating: Inclusion criteria and method of recruitment seem reasonable and appropriate, but the study has used registry data for the prognostic model			
**B. Applicability**			
Describe included participants, setting and dates: The data from Maharaj Nakorn Chiang Mai Hospital (PCI-capable hospital) STEMI registry during the period 2007–2012 were analysed.			
**Concern that the included participants and setting do not match the review question**	**CONCERN:**	**LOW**	**LOW**
Rationale of applicability rating: Description of included participants, setting and dates seem reasonable to the review question.			
**DOMAIN 2: Predictors**			
**A. Risk of Bias**			
List and describe predictors included in the final model, e.g. definition and timing of assessment:			
		Dev	Val
2.13 Were predictors defined and assessed in a similar way for all participants?			**Y**
2.14 Were predictor assessments made without knowledge of outcome data?			**PY**
2.15 Are all predictors available at the time the model is intended to be used?			**Y**
**Risk of bias introduced by predictors or their assessment**	**RISK:**		**LOW**
Rationale of bias rating: Predictors used in this study together with their assessment seem reasonable and low risk of bias.			
**B. Applicability**			
Concern that the definition, assessment or timing of predictors in the model do not match the review question	**CONCERN:**		**LOW**
Rationale of applicability rating: The definition, assessment and timing of predictors in the model seem reasonable to the review question.			

**DOMAIN 3: Outcome**		
**A. Risk of Bias**		
Describe the outcome, how it was defined and determined, and the time interval between predictor assessment and outcome determination: The outcomes of the study were 30-day and 6-month composite cardiovascular outcome including death, re-hospitalisation with acute coronary syndrome (ACS), re-hospitalisation with heart failure (HF) and stroke at 30-day and 6-month composite cardiovascular outcome.		
	Dev	Val
3.25 Was the outcome determined appropriately?		**Y**

3.26 Was a pre-specified or standard outcome definition used?		**Y**
3.27 Were predictors excluded from the outcome definition?		**PY**
3.28 Was the outcome defined and determined in a similar way for all participants?		**Y**
3.29 Was the outcome determined without knowledge of predictor information?		**PY**
3.30 Was the time interval between predictor assessment and outcome determination appropriate?		**Y**
**Risk of bias introduced by the outcome or its determination** **RISK:**		**LOW**

Rationale of bias rating:
As this study extracted registry data, the determination of outcome is at low risk of bias.

B. Applicability

At what time point was the outcome determined:
The outcomes of the study were 30-day and 6-month composite cardiovascular outcome including death, re-hospitalisation with acute coronary syndrome (ACS), re-hospitalisation with heart failure (HF) and stroke at 30-day and 6-month composite cardiovascular outcome.

**Concern that the outcome, its definition, timing or determination do not match the review question**	**CONCERN:**		**LOW**

Rationale of applicability rating:
The outcome, its definition, timing and its determination seem reasonable to the review question.

DOMAIN 4: Analysis

Risk of Bias

Describe numbers of participants, number of candidate predictors, outcome events and events per candidate predictor:

	Low GRACE score group	Intermediate-high GRACE score group
**Number of events**	In-hospital mortality= 1(0.4%)<br>At 30 days = 5 (2.2%)<br>At 6 months = 9 (3.9%)	In-hospital mortality= 2(1.8%)<br>At 30 days = 13 (11.6%)<br>At 6 months = 15 (13.4%)
**Number of participants**	229	112

Describe how the model was developed (for example in regard to modelling technique (e.g. survival or logistic modelling), predictor selection, and risk group definition):
Not applicable

Describe whether and how the model was validated, either internally (e.g. bootstrapping, cross validation, random split sample) or externally (e.g. temporal validation, geographical validation, different setting, different type of participants):
Not applicable

Describe the performance measures of the model, e.g. (re)calibration, discrimination, (re)classification, net benefit, and whether they were adjusted for optimism:
At 6 months: AuROC 0.7464 (95%CI: 0.698 – 0.793)

Describe any participants who were excluded from the analysis:
Not reported

Describe missing data on predictors and outcomes as well as methods used for missing data:
Not reported

	Dev	Val
4.37 Were there a reasonable number of participants with the outcome?		N
4.38 Were continuous and categorical predictors handled appropriately?		Y
4.39 Were all enrolled participants included in the analysis?		PY
4.40 Were participants with missing data handled appropriately?		NI
4.41 Was selection of predictors based on univariable analysis avoided?		
4.42 Were complexities in the data (e.g. censoring, competing risks, sampling of controls) accounted for appropriately?		NI
4.43 Were relevant model performance measures evaluated appropriately?		Y
4.44 Were model overfitting and optimism in model performance accounted for?		
4.45 Do predictors and their assigned weights in the final model correspond to the results from multivariable analysis?		
**Risk of bias introduced by the analysis** **RISK:**		**HIGH**

Rationale of bias rating:
There were inadequate information on handling of missing data. Additionally, the number of events per predictor is low.

Overall judgement about risk of bias and applicability of the prediction model evaluation

Overall judgement of risk of bias	RISK:	HIGH
Summary of sources of potential bias: The sources of potential bias for two domains were at low risk except for the participants and analysis domains.		
**Overall judgement of applicability**	**CONCERN:**	**LOW**
Summary of applicability concerns: The overall applicability of the study participants, predictors and outcomes were of all low concern.		

6 Selvarajah 2012

**Classify the evaluation based on its aim**			
**Type of prediction study**	**PROBAST boxes to complete**	**Tick as appropriate**	**Definition for type of prediction model study**
Development only	Development		Prediction model development without external validation. These studies may include internal validation methods, such as bootstrapping and cross-validation techniques.
Development and validation	Development and validation		Prediction model development combined with external validation in other participants in the same article.
Validation only	Validation	✓	External validation of existing (previously developed) model in other participants.

**Publication reference**	Selvarajah S, Fong AYY, Selvaraj G, Haniff J, Uiterwaal CSPM, Bots ML. An Asian validation of the TIMI risk score for ST-segment elevation myocardial infarction. PloS one. 2012;7(7):e40249-e.
**Models of interest**	TIMI score
**Outcome of interest**	30 days mortality

**DOMAIN 1: Participants**			
**A. Risk of Bias**			
Describe the sources of data and criteria for participant selection: "The National Cardiovascular Disease Database (NCVD) in Malaysia is an on-going observational prospective registry of patients who present with ACS. It commenced on the 1st of January 2006. Patient recruitment occurs at 16 hospitals with varying facilities; 14 from the Ministry of Health, 1 university hospital and the National Heart Institute of Malaysia… This study made use of anonymized data from patients who presented with STEMI registered from 1st January 2006 till 31st December 2008 with follow up details recorded till 31st December 2009"			
		Dev	Val
1.11 Were appropriate data sources used, e.g. cohort, RCT or nested case-control study data?			**N**
1.12 Were all inclusions and exclusions of participants appropriate?			**Y**
**Risk of bias introduced by selection of participants**	**RISK:**		**HIGH**
Rationale of bias rating: Participant data were obtained from an existing database			
**B. Applicability**			
Describe included participants, setting and dates: This study made use of anonymized data from patients who presented with STEMI registered from 1st January 2006 till 31st December 2008 with follow up details recorded till 31st December 2009"			
**Concern that the included participants and setting do not match the review question**	**CONCERN:**		**LOW**
Rationale of applicability rating: Included participants and setting were reasonable for the review question.			

**DOMAIN 2: Predictors**
**A. Risk of Bias**
List and describe predictors included in the final model, e.g. definition and timing of assessment: "The TIMI risk score for STEMI was developed using the study population from the Intravenous nPA for Treatment of Infarcting Myocardium Early II (InTIME II) trial [13]. The study population of the InTIME II trial will be referred to as the 'TIMI development' population for this study. The elements of the TIMI risk score are age, systolic blood pressure, heart rate, Killip classification, infarct location or left bundle branch block, history of diabetes, hypertension or angina pectoris, weight and time to treatment. The TIMI STEMI scoring mechanism has been published [6]. For this study, the TIMI risk score is slightly modified for

14

'time to treatment' variable. Time to treatment is defined as time from presentation (not symptom onset) to reperfusion, either via thrombolytics (door-to-needle time) or primary percutaneous coronary intervention (door-to-balloon time). Those who did not receive reperfusion therapy for the following reasons; missed thrombolysis (12.6%), thrombolysis was contraindicated (4%) or patient refused treatment (0.2%), were given a score of 1 for time to treatment. "			
		Dev	Val
2.16 Were predictors defined and assessed in a similar way for all participants?			PY
2.17 Were predictor assessments made without knowledge of outcome data?			PY
2.18 Are all predictors available at the time the model is intended to be used?			Y
**Risk of bias introduced by predictors or their assessment**	**RISK:**		**LOW**
Rationale of bias rating:<br>In view of the usage of a registry database for the participant predictors assessment, we believe that there is low risk of bias in this domain.			
**B. Applicability**			
Concern that the definition, assessment or timing of predictors in the model do not match the review question	**CONCERN:**		**LOW**
Rationale of applicability rating:<br>Definition, assessment and timing of predictors in the model seem reasonable and acceptable to the review question.			

**DOMAIN 3: Outcome**			
**A. Risk of Bias**			
Describe the outcome, how it was defined and determined, and the time interval between predictor assessment and outcome determination:<br>"The outcome of interest was 30-day mortality. Details on mortality were obtained via hospital records and a 30-day follow up phone call to the patient/relatives. Confirmation of mortality is done yearly via record linkages with the Malaysian National Registration Department for deaths in the country".			
		Dev	Val
3.31 Was the outcome determined appropriately?			PY
3.32 Was a pre-specified or standard outcome definition used?			Y
3.33 Were predictors excluded from the outcome definition?			Y
3.34 Was the outcome defined and determined in a similar way for all participants?			PY
3.35 Was the outcome determined without knowledge of predictor information?			PY
3.36 Was the time interval between predictor assessment and outcome determination appropriate?			Y
**Risk of bias introduced by the outcome or its determination**	**RISK:**		**LOW**
Rationale of bias rating:<br>The description and determination of outcomes are deemed reasonable and acceptable.			
**B. Applicability**			
At what time point was the outcome determined:<br>"The outcome of interest was 30-day mortality."			
**Concern that the outcome, its definition, timing or determination do not match the review question**	**CONCERN:**		**LOW**
Rationale of applicability rating:<br>The outcome, its definition, timing and determination seem reasonable and acceptable to the review question.			

**DOMAIN 4: Analysis**
**Risk of Bias**
Describe numbers of participants, number of candidate predictors, outcome events and events per candidate predictor:<br>Number of participants: Overall 4,701; Diabetes subgroup: 1,707; and Renal impairment subgroup: 156<br>Number of candidate predictors: 8<br>Outcome events: Overall 11.1%; diabetes subgroup 14%; and renal impairment subgroup 27.6%<br>Events per candidate predictor: Overall 57; diabetes subgroup 26; and renal impairment subgroup 5
Describe how the model was developed (for example in regard to modelling technique (e.g. survival or logistic modelling), predictor selection, and risk group definition):<br>Not applicable
Describe whether and how the model was validated, either internally (e.g. bootstrapping, cross validation, random split sample) or externally (e.g. temporal validation, geographical validation, different setting, different type of participants):<br>Temporal and geographical validation
Describe the performance measures of the model, e.g. (re)calibration, discrimination, (re)classification, net benefit, and whether they were adjusted for optimism:

	Overall population	Diabetes subgroup	Renal impairment subgroup
Calibration results	Chi square of goodness of fit test: 0.936	Chi square of goodness of fit test: 0.983	Chi square of goodness of fit test: 0.006
Discrimination results	C-statistic: 0.785 (95CI: 077 – 0.81)	C statistic: 0.764 (95%CI: 0.73 – 0.80)	C statistic: 0.761 (95%CI: 0.68 – 0.85)

Describe any participants who were excluded from the analysis:
Not reported

Describe missing data on predictors and outcomes as well as methods used for missing data:
"Missing data was checked to determine if it was Missing At Random using the separate variance t test. Seven variables with missing values were imputed. Those with missing values of ,5% (age 0.1%, systolic blood pressure 1.1%, heart rate at presentation 2%, sex 2.2% and smoking status 4.9%) were imputed using mean or median values where applicable. Two variables with .5% missing (time to treatment, 23.9% and weight, 36.1%) were imputed using single imputation with a random error term method."

		Dev	Val
4.46 Were there a reasonable number of participants with the outcome?			N
4.47 Were continuous and categorical predictors handled appropriately?			Y
4.48 Were all enrolled participants included in the analysis?			Y
4.49 Were participants with missing data handled appropriately?			Y
4.50 Was selection of predictors based on univariable analysis avoided?			
4.51 Were complexities in the data (e.g. censoring, competing risks, sampling of controls) accounted for appropriately?			NI
4.52 Were relevant model performance measures evaluated appropriately?			Y
4.53 Were model overfitting and optimism in model performance accounted for?			
4.54 Do predictors and their assigned weights in the final model correspond to the results from multivariable analysis?		NI	
**Risk of bias introduced by the analysis**	**RISK:**		**HIGH**

Rationale of bias rating:
We judged this domain as high risk of bias due to the insufficient number of events per predictor in the renal impairment subgroup.

**Overall judgement about risk of bias and applicability of the prediction model evaluation**		
**Overall judgement of risk of bias**	**RISK:**	**HIGH**
Summary of sources of potential bias: Overall potential source of bias is judged as high risk due to concerns in the participants and analysis domains		
**Overall judgement of applicability**	**CONCERN:**	**LOW**
Summary of applicability concerns: The overall applicability of the study participants, predictors and outcomes were of all low concern.		

7 MARTHA 2021

**Classify the evaluation based on its aim**			
**Type of prediction study**	**PROBAST boxes to complete**	**Tick as appropriate**	**Definition for type of prediction model study**
Development only	Development		Prediction model development without external validation. These studies may include internal validation methods, such as bootstrapping and cross-validation techniques.
Development and validation	Development and validation		Prediction model development combined with external validation in other participants in the same article.
Validation only	Validation	✓	External validation of existing (previously developed) model in other participants.

**Publication reference**	Martha JW, Sihite TA, Listina D. The Difference in Accuracy Between Global Registry of Acute Coronary Events Score and Thrombolysis in Myocardial Infarction Score in Predicting In-Hospital Mortality of Acute ST-Elevation Myocardial Infarction Patients. Cardiol Res. 2021;12(3):177-85.
**Models of interest**	GRACE score and TIMI score
**Outcome of interest**	30 days mortality

16

DOMAIN 1: Participants			
**A. Risk of Bias**			
Describe the sources of data and criteria for participant selection:<br>"This was an observational study with a retrospective cohort. Data were collected consecutively from the medical records of patients diagnosed with STEMI or with the ICD code of I21.0-I21.3 from July 2018 to June 2019 and included patient's history, physical examination, laboratory results, electrocardiogram (ECG) record, TIMI score, GRACE score and reperfusion time. There were 255 of 341 patients with a medical record code of I21.0-I21.3 who met the inclusion criteria. Patients with I21.0-I21.3 code but with a diagnosis other than STEMI, such as NSTEACS and occlusion myocardial infarction (OMI), and those with incomplete or absent medical re- cords, were excluded. "			
		Dev	Val
1.13 Were appropriate data sources used, e.g. cohort, RCT or nested case-control study data?			**N**
1.14 Were all inclusions and exclusions of participants appropriate?			**Y**
**Risk of bias introduced by selection of participants**	**RISK:**		**HIGH**
Rationale of bias rating:<br>Participant data were obtained retrospectively from hospital medical records.			
**B. Applicability**			
Describe included participants, setting and dates:<br>"This was an observational study with a retrospective cohort. Data were collected consecutively from the medical records of patients diagnosed with STEMI or with the ICD code of I21.0-I21.3 from July 2018 to June 2019"			
**Concern that the included participants and setting do not match the review question**	**CONCERN:**		**LOW**
Rationale of applicability rating:<br>Included participants and setting were reasonable for the review question.			

DOMAIN 2: Predictors			
**A. Risk of Bias**			
List and describe predictors included in the final model, e.g. definition and timing of assessment:<br>No defined			
		Dev	Val
2.19 Were predictors defined and assessed in a similar way for all participants?			NI
2.20 Were predictor assessments made without knowledge of outcome data?			PY
2.21 Are all predictors available at the time the model is intended to be used?			PY
**Risk of bias introduced by predictors or their assessment**	**RISK:**		**UNCLEAR**
Rationale of bias rating:<br>The predictors assessed and its timing of assessment were not clearly defined.			
**B. Applicability**			
Concern that the definition, assessment, or timing of predictors in the model do not match the review question	**CONCERN:**		**UNCLEAR**
Rationale of applicability rating:<br>Definition, assessment and timing of predictors in the models were not clear.			

DOMAIN 3: Outcome			
**A. Risk of Bias**			
Describe the outcome, how it was defined and determined, and the time interval between predictor assessment and outcome determination:<br>*"*This study was expected to identify the difference in accuracy between the GRACE score and TIMI score in predicting in-hospital mortality of STEMI"			
		Dev	Val
3.37 Was the outcome determined appropriately?			NI
3.38 Was a pre-specified or standard outcome definition used?			NI
3.39 Were predictors excluded from the outcome definition?			PY
3.40 Was the outcome defined and determined in a similar way for all participants?			PY
3.41 Was the outcome determined without knowledge of predictor information?			PY
3.42 Was the time interval between predictor assessment and outcome determination appropriate?			Y
**Risk of bias introduced by the outcome or its determination**	**RISK:**		**UNCLEAR**

Rationale of bias rating:<br>There was inadequate information on the determination and assessment of outcomes.			
**B. Applicability**			
At what time point was the outcome determined:<br>*"*This study was expected to identify the difference in accuracy between the GRACE score and TIMI score in predicting in-hospital mortality of STEMI"			
**Concern that the outcome, its definition, timing, or determination do not match the review question**	**CONCERN:**		**LOW**
Rationale of applicability rating:<br>The outcome seems reasonable and acceptable to the review question.			

**DOMAIN 4: Analysis**		
**Risk of Bias**		
Describe numbers of participants, number of candidate predictors, outcome events and events per candidate predictor:<br>Number of participants: 255<br>Number of candidate predictors: TIMI 8, GRACE 8<br>Outcome events: 45<br>Events per candidate predictor: 6 for TIMI and 6 for GRACE		
Describe how the model was developed:<br>Not applicable		
Describe whether and how the model was validated, either internally or externally:<br>Temporal and geographical validation		
Describe the performance measures of the model		

Model	Calibration measure	Discrimination measure
TIMI score	Not reported	0.92 (0.88 – 0.95)
GRACE score	Not reported	0.84 (0.78 – 0.88)

Describe any participants who were excluded from the analysis:<br>Not reported		
Describe missing data on predictors and outcomes as well as methods used for missing data:<br>Not defined		
	Dev	Val
4.55 Were there a reasonable number of participants with the outcome?		N
4.56 Were continuous and categorical predictors handled appropriately?		NI
4.57 Were all enrolled participants included in the analysis?		PY
4.58 Were participants with missing data handled appropriately?		NI
4.59 Was selection of predictors based on univariable analysis avoided?		
4.60 Were complexities in the data (e.g. censoring, competing risks, sampling of controls) accounted for appropriately?		NI
4.61 Were relevant model performance measures evaluated appropriately?		Y
4.62 Were model overfitting and optimism in model performance accounted for?		
4.63 Do predictors and their assigned weights in the final model correspond to the results from multivariable analysis?	NI	
**Risk of bias introduced by the analysis** **RISK:**		**HIGH**
Rationale of bias rating:<br>We judged this domain as high risk of bias due to the insufficient number of events per predictor in both models		

**Overall judgement about risk of bias and applicability of the prediction model evaluation**		
**Overall judgement of risk of bias**	**RISK:**	**HIGH**
Summary of sources of potential bias:<br>Overall potential source of bias is judged as high risk due to concerns in the participants and analysis domains		
**Overall judgement of applicability**	**CONCERN:**	**LOW**
Summary of applicability concerns:<br>The overall applicability of the study participants, predictors and outcomes were of all low concern.		
