# Supplementary material for: Systematic review and meta-analysis of prognostic models in Southeast Asian populations with acute myocardial infarction
Source: Front Cardiovasc Med. 2022 Jul 26;9:921044. doi: 10.3389/fcvm.2022.921044 (PMC9360484; doi:10.3389/fcvm.2022.921044)
Supplement: Supplementary file 4 [file Data_Sheet_4.PDF]

Supplementary File 4

R software script for meta-analysis

Meta-analysis and forest plot of GRACE risk score

```
library(metamisc)

library(metafor)

forestPlot <- read_excel("forestPlot.xlsx", +  sheet = "GRACE_Val")

par (cex=1.0, font=1)
forest(forestPlot$Cstat, ci.lb=forestPlot$Cstat95LB,ci.ub=forestPlot$Cstat95UB,
        slab=forestPlot$study, xlab = "C-statistic", refline = 0.5, cex = 1.0,
        alim=(c(0.4,1)),steps=5,xlim=(c(-0.0,1.5)), psize=1.0,
        ylim=c(1,nrow(forestPlot)+5),rows=c(3:(nrow(forestPlot)+2)))


fit1 <- with(forestPlot, valmeta(cstat=Cstat, cstat.se=CstatSE, N=Total, O=Events, slab=study))


par(cex=1.0, font=1)
addpoly(x = fit1$est, ci.lb=fit1$ci.lb, ci.ub=fit1$ci.ub, row=2)


par(cex=1.0, font=1)
text(1.1,c(3:(nrow(forestPlot)+2)),forestPlot$Events,pos=2)
text(1.1,c(3:(nrow(forestPlot)+2)),forestPlot$Total,pos=4)
text(1.12,c((nrow(forestPlot)+2):3),rep("/",nrow(forestPlot)),pos=2)


par(cex=1.0, font=2)
text(c(0,1.1,1.12,1.1,1.5),nrow(forestPlot)+3.5, c("Study","Events","/","Total","C-statistic [95%
CI]"), pos=c(4,2,2,4,2))
text(0,c(2), c("Pooled estimate"), pos=4)
```
