# Supplementary material for: Systematic review and meta-analysis of prognostic models in Southeast Asian populations with acute myocardial infarction
Source: Front Cardiovasc Med. 2022 Jul 26;9:921044. doi: 10.3389/fcvm.2022.921044 (PMC9360484; doi:10.3389/fcvm.2022.921044)
Supplement: Supplementary file 5 [file Data_Sheet_5.PDF]

Supplementary File 5

List of predictors included in developed models

Model	SVMvarImp-SBE-SVM	SVMvarImp-SBE-SVM	SVMvarImp-SBE-SVM	SMIR	Singapore score
Outcome	In-hospital mortality	30-day mortality	1-year mortality	In-hospital cardiac mortality, 30-day cardiac mortality, 1-year cardiac mortality, and 1-year hospitalisation for heart failure	In-hospital mortality
Predictors	Age	Age	Age	Age	Age
	Beta blockers	Aspirin	ACE inhibitor	Creatinine	Cardiac arrest
	Cardiac catheterization	Beta blockers	Diabetes	Haemoglobin	Cardiac enzymes
	Chronic renal disease	Cardiac catheterization	Diuretics	History of diabetes	Creatinine
	Diastolic blood pressure	Diuretics	Fasting blood glucose	History of ischaemic heart disease	Killip class
	Diuretics	Family history of premature CVD	Heart rate	Ischaemic Time	Pulse rate
	ECG- location lateral lead	Fasting blood glucose	Hypertension	Killip class	ST deviation
	ECG-type bundle branch block	HDL	Insulin	LVEF during hospitalisation	Systolic blood pressure
	Family history of premature CVD	Heart rate	Killip class	Troponin T/I	
	Fasting blood glucose	Insulin	Percutaneous coronary intervention		
	Heart rate	Killip class	Smoking status		
	Killip class	Percutaneous coronary intervention	Systolic blood pressure		
	Statin	Race			
	Systolic blood pressure				
	Oral hypoglycaemic agent.				
